# Likelihood analysis of the pMSSM11 in light of LHC 13-TeV data

**DOI:** 10.1140/epjc/s10052-018-5697-0

**Published:** 2018-03-24

**Authors:** E. Bagnaschi, K. Sakurai, M. Borsato, O. Buchmueller, M. Citron, J. C. Costa, A. De Roeck, M. J. Dolan, J. R. Ellis, H. Flächer, S. Heinemeyer, M. Lucio, D. Martínez Santos, K. A. Olive, A. Richards, V. C. Spanos, I. Suárez Fernández, G. Weiglein

**Affiliations:** 1 DESY, Notkestraße 85, 22607 Hamburg, Germany; 2 Faculty of Physics, Institute of Theoretical Physics, University of Warsaw, ul. Pasteura 5, 02-093 Warsaw, Poland; 3 Instituto Galego de Física de Altas Enerxías, Universidade de Santiago de Compostela, Santiago de Compostela, Spain; 4 High Energy Physics Group, Blackett Laboratory, Imperial College, Prince Consort Road, London, SW7 2AZ UK; 5 Experimental Physics Department, CERN, 1211 Geneva 23, Switzerland; 6 Antwerp University, 2610 Wilrijk, Belgium; 7 ARC Centre of Excellence for Particle Physics at the Terascale, School of Physics, University of Melbourne, 3010 Melbourne, Australia; 8 Theoretical Particle Physics and Cosmology Group, Department of Physics, King’s College London, London, WC2R 2LS UK; 9 National Institute of Chemical Physics and Biophysics, Rävala 10, 10143 Tallinn, Estonia; 10 Theoretical Physics Department, CERN, 1211 Geneva 23, Switzerland; 11 H.H. Wills Physics Laboratory, University of Bristol, Tyndall Avenue, Bristol, BS8 1TL UK; 12 Campus of International Excellence UAM+CSIC, Cantoblanco, 28049 Madrid, Spain; 13 Instituto de Física Teórica UAM-CSIC, C/ Nicolas Cabrera 13-15, 28049 Madrid, Spain; 14 Instituto de Física de Cantabria (CSIC-UC), Avda. de Los Castros s/n, 39005 Santander, Spain; 15 William I. Fine Theoretical Physics Institute, School of Physics and Astronomy, University of Minnesota, Minneapolis, MN 55455 USA; 16 Section of Nuclear and Particle Physics, Department of Physics, National and Kapodistrian University of Athens, 15784 Athens, Greece

## Abstract

We use MasterCode to perform a frequentist analysis of the constraints on a phenomenological MSSM model with 11 parameters, the pMSSM11, including constraints from $\sim 36$/fb of LHC data at 13 TeV and PICO, XENON1T and PandaX-II searches for dark matter scattering, as well as previous accelerator and astrophysical measurements, presenting fits both with and without the $(g-2)_\mu$ constraint. The pMSSM11 is specified by the following parameters: 3 gaugino masses $M_{1,2,3}$, a common mass for the first-and second-generation squarks $m_{\tilde{q}}$ and a distinct third-generation squark mass $m_{\tilde{q}_3}$, a common mass for the first-and second-generation sleptons $m_{\tilde{\ell }}$ and a distinct third-generation slepton mass $m_{\tilde{\tau }}$, a common trilinear mixing parameter *A*, the Higgs mixing parameter $\mu$, the pseudoscalar Higgs mass $M_A$ and $\tan \beta$. In the fit including $(g-2)_\mu$, a Bino-like $\tilde{\chi }^0_{1}$ is preferred, whereas a Higgsino-like $\tilde{\chi }^0_{1}$ is mildly favoured when the $(g-2)_\mu$ constraint is dropped. We identify the mechanisms that operate in different regions of the pMSSM11 parameter space to bring the relic density of the lightest neutralino, $\tilde{\chi }^0_{1}$, into the range indicated by cosmological data. In the fit including $(g-2)_\mu$, coannihilations with $\tilde{\chi }^0_{2}$ and the Wino-like $\tilde{\chi }^\pm _{1}$ or with nearly-degenerate first- and second-generation sleptons are active, whereas coannihilations with the $\tilde{\chi }^0_{2}$ and the Higgsino-like $\tilde{\chi }^\pm _{1}$ or with first- and second-generation squarks may be important when the $(g-2)_\mu$ constraint is dropped. In the two cases, we present $\chi ^2$ functions in two-dimensional mass planes as well as their one-dimensional profile projections and best-fit spectra. Prospects remain for discovering strongly-interacting sparticles at the LHC, in both the scenarios with and without the $(g-2)_\mu$ constraint, as well as for discovering electroweakly-interacting sparticles at a future linear $e^+ e^-$ collider such as the ILC or CLIC.

## Introduction

Supersymmetric (SUSY) models of TeV-scale physics are being subjected to increasing pressure by the strengthening constraints imposed by LHC experiments [1, 2] and searches for Dark Matter (DM) [3–6]. In particular, in the context of models with soft supersymmetry-breaking parameters constrained to be universal at a high unification scale, the LHC limits on sparticle masses have been in increasing tension with a supersymmetric interpretation of the anomalous magnetic moment of the muon, $(g-2)_\mu$, which would require relatively light sleptons and electroweak gauginos [7–13]. This pressure has been ratcheted up by the advent of $\sim 36$/fb of data from Run 2 of the LHC at a centre-of-mass energy of 13 TeV [14–16],^1^ which probe supersymmetric models at significantly higher mass scales than was possible in Run 1 at 7 and 8 TeV in the centre of mass. In parallel, direct searches for DM scattering have also been making significant progress towards the neutrino ‘floor’ [17, 18], in particular with the recent data releases from the LUX, PICO, XENON1T and PandaX-II experiments [3–6]. Here we analyze these constraints in the minimal supersymmetric extension of the Standard Model (MSSM), which, because of *R*-parity, has a stable cosmological relic particle that we assume to be the lightest neutralino, $\tilde{\chi }^0_{1}$ [19, 20].

The strengthening phenomenological, experimental and astrophysical constraints on supersymmetry (SUSY) were initially explored mainly in the contexts of models in which SUSY breaking was assumed to be universal at the GUT scale, such as the constrained MSSM (CMSSM) [7–11, 21–31], non-universal Higgs models (NUHM1,2) [11, 12],^2^ the minimal anomaly-mediated SUSY-breaking model (mAMSB) [33], and models based on the SU(5) group [34]. These models are tractable by virtue of having a relatively limited number of parameters, though the universality assumptions they employ are not necessarily well supported in scenarios motivated by fundamental principles, such as string theory. Their limited parameter spaces are amenable to analysis, e.g., in the frequentist approach we follow, in which one constructs a global likelihood function that embodies all the information provided by the multiple constraints.

Alternatively, one may study phenomenological models in which the soft SUSY-breaking parameters are not constrained by any universality assumptions, though subject to milder constraints emanating, in particular, from upper limits on SUSY contributions to flavour-changing processes. These phenomenological MSSM (pMSSM) [35–43] models contain many more parameters, whose exploration is computationally demanding. There have been cut-based global analyses of variants of the pMSSM with as many as 19 parameters [44–48] and global fits focused on specific sectors or parameter ranges [49, 50], however in the past we have restricted our frequentist attentions to a variant of the pMSSM with 10 parameters, the pMSSM10 [13, 51]. These were taken to be 3 independent gaugino masses, $M_{1,2,3}$, a common electroweak-scale mass for the first-and second-generation squarks, $m_{\tilde{q}}$, a distinct mass for the third-generation squarks, $m_{\tilde{q}_3}$, a common electroweak-scale mass $m_{\tilde{l}}$ for the sleptons, a single trilinear mixing parameter *A* that is universal at the electroweak scale, the Higgs mixing parameter $\mu$, the pseudoscalar Higgs mass, $M_A$ and the ratio of Higgs vevs, $\tan \beta$.^3^

It is desirable to extend this type of analysis to more general variants of the pMSSM, for a couple of reasons. One is that the lower bounds on sparticle masses will, in general, be weaker in models with more parameters, so one should explore such models before making statements about the magnitudes of these lower bounds and prospects for discovering sparticles at the LHC or elsewhere. Another reason is that reconciling the strengthening LHC constraints with the cosmological DM density constraint requires, in general, specific relations between sparticle masses that suppress the relic density via coannihilation effects and/or rapid annihilations through direct-channel resonances. Therefore one should study models capable of accommodating these DM mechanisms [51].

**Table 1:** The ranges of the pMSSM11 parameters sampled, which are divided into the indicated numbers of segments, yielding the total number of sample boxes shown in the last row. In the last column, we indicate the kind of prior used, where “soft” means a flat prior with Gaussian tails

Parameter	Range	Number of segments	Prior type
$M_1$	(−4, 4)$\,\mathrm {TeV}$	6	Soft
$M_2$	(0, 4)$\,\mathrm {TeV}$	2	Soft
$M_3$	(−4, 4)$\,\mathrm {TeV}$	4	Soft
$m_{\tilde{q}}$	(0, 4)$\,\mathrm {TeV}$	2	Soft
$m_{\tilde{q}_3}$	(0, 4)$\,\mathrm {TeV}$	2	Soft
$m_{\tilde{\ell }}$	(0, 2)$\,\mathrm {TeV}$	1	Soft
$m_{\tilde{\tau }}$	(0, 2)$\,\mathrm {TeV}$	1	Soft
$M_A$	(0, 4)$\,\mathrm {TeV}$	2	Soft
*A*	(−5, 5)$\,\mathrm {TeV}$	1	Soft
$\mu$	(−5, 5)$\,\mathrm {TeV}$	1	Soft
$\tan \beta$	(1, 60)	1	Soft
$M_t$ [55]	$\mu = 173.34$ GeV, $\sigma = 0.76$ GeV	1	Gaussian
$M_Z$ [56]	$\mu = 91.1876$ GeV, $\sigma = 0.0021$ GeV	1	Gaussian
$\Delta \alpha ^{(5)}_{\mathrm {had}}(M_Z)$ [56]	$\mu = 0.02771$, $\sigma = 0.00011$	1	Gaussian
Total number of boxes		384	

Examples of DM mechanisms that have been studied extensively in the past [51] include coannihilation with the lighter stau slepton, $\tilde{\tau }_1$, the lighter chargino, $\tilde{\chi }^\pm _{1}$, or the lighter stop squark, $\tilde{t}_{1}$, and rapid annihilations via the *Z* boson, the 125-GeV Higgs boson, *h*, or the heavier MSSM Higgs bosons, *H* / *A*. More recently, the possibility of coannihilation with gluinos, ${\tilde{g}}$, has been explored in models with non-universal gaugino masses [53, 54], and coannihilation with the right-handed up-type squarks of the first two generations, ${\tilde{u}_R}/{\tilde{c}_R}$, emerged as a possibility in an SU(5) model with non-universal scalar masses $m_5, m_{10}$ for sfermions in ${\bar{\mathbf {5}}}$ and $\mathbf {10}$ representations [34].

All of these were possibilities in the pMSSM10, but in that scenario the stau and smuon masses were fixed to be equal, putting the LHC constraints on stau coannihilation in tension with the possibility of a SUSY interpretation of $(g-2)_\mu$, a tension that has increased with the advent of the first LHC data at 13 TeV. In this paper we study two possible resolutions of this issue. We study an extension of the parameter space of the pMSSM10 to 11 parameters by relaxing the equality between the soft SUSY-breaking contributions to the stau mass and to the (still common) masses of the smuon and selectron, the pMSSM11. In order to assess the importance of the $(g-2)_\mu$ constraint, we also consider a fit omitting the SUSY interpretation of $(g-2)_\mu$. The principal results of this paper are comparisons between the likelihoods of different spectra in the pMSSM11 with and without $(g-2)_\mu$, and comparisons between the likelihoods of different DM mechanisms including $\tilde{\tau }_1$, ${\tilde{\ell }}$, ${\tilde{q}}$ and ${\tilde{g}}$ coannihilation, highlighting the impacts of the LHC 13 TeV and recent DM scattering data.

The layout of this paper is as follows. In Sect. 2 we specify the framework of our analysis. Section 2.1 specifies the pMSSM11, establishes our notation for its parameters and describes our procedure for sampling the pMSSM11 parameter space. In Sect. 2.2 we review the MasterCode tool to construct a global $\chi ^2$ likelihood function combining constraints on model parameters, Sect. 2.3 describes our treatments of the electroweak and flavour constraints, including some updates compared with our previous analyses. In Sect. 2.4 we give details on our DM analysis, which includes constraints on both spin-independent and -dependent DM scattering [3–6]. Our implementations of the constraints from $\sim 36$/fb of LHC at 13 TeV [14–16] are discussed in Sect. 2.5. Then, in Sect. 3.1 we present results for the global likelihood function in various parameter planes, highlighting the regions where different DM mechanisms operate and comparing results with and without the $(g-2)_\mu$ constraint being applied. Section 4 displays the one-dimensional profile likelihood functions for various masses, mass differences and other observables in these two cases, and also shows predictions for spin-independent and -dependent DM scattering. Section 5 highlights the impacts of the LHC 13-TeV data [14–16] and the recent direct searches for astrophysical DM [3–6]. Section 6 discusses the best-fit points, favoured and allowed spectra in these pMSSM scenarios. Finally, Sect. 7 summarizes our conclusions.

## Analysis framework

### Model parameters

As mentioned above, in this paper we consider a pMSSM scenario with eleven parameters, namely

1 $$\begin{aligned} \begin{aligned} \mathrm{3~gaugino~masses}:&\; M_{1,2,3}, \\ \mathrm{2~squark~masses}:&\; {m_{\tilde{q}}\, \equiv } \, m_{\tilde{q}_1}, m_{\tilde{q}_2} \\&\ne \, m_{\tilde{q}_3}\, = \, m_{\tilde{t}}, m_{\tilde{b}},\\ \mathrm{2~slepton~masses}:&\; {m_{\tilde{\ell }}} \equiv m_{\tilde{\ell }_1} = m_{\tilde{\ell }_2} = m_{\tilde{e}_{,}} m_{\tilde{\mu }_{1}}\\&\ne \, m_{\ell _3} \, = \, m_{\tilde{\tau }}, \\ \mathrm{1~trilinear~coupling}:&\; A, \\ \mathrm{Higgs~mixing~parameter}:&\; \mu , \\ \mathrm{pseudoscalar~Higgs~mass}:&\; M_A, \\ \mathrm{ratio~of~vevs}:&\; \tan \beta , \end{aligned} \end{aligned}$$

where $q_{1,2} \equiv u, d, s, c$, we assume soft SUSY-breaking parameters for left- and right-handed sfermions, and the sneutrinos have the same soft SUSY-breaking parameter as the corresponding charged sfermions. All of these parameters are specified at a renormalisation scale $M_\mathrm{SUSY}$ given by the geometric mean of the masses of the scalar top eigenstates, $M_\mathrm{SUSY}\equiv \sqrt{m_{\tilde{t}_{1}} m_{\tilde{t}_{2}}}$, which is also the scale at which electroweak symmetry breaking conditions are imposed. We allow the sign of the mixing parameter $\mu$ to be either positive or negative. The important difference from the pMSSM10 scenario we studied previously [13] is that the first- and second-generation slepton mass $m_{\tilde{\ell }}$ and the stau mass $m_{\tilde{\tau }}$ are decoupled in the pMSSM11.^4^

The ranges of these parameters sampled in our analysis are displayed in Table 1. In each case, we indicate in the third column of Table 1 how the ranges of most of these parameters are divided into segments, much as we did previously for our analysis of the pMSSM10 [13].

These segments define boxes in the eleven-dimensional parameter space, which we sample using the MultiNest package [57–59]. In order to ensure a smooth overlap between boxes and eliminate features associated with their boundaries, we choose for each box a prior such that 80% of the sample has a flat distribution within the nominal box, and 20% of the sample is in normally-distributed tails extending outside the box. An initial scan over all mass parameters with absolute values $\le 4 \,\mathrm {TeV}$ showed that non-trivial behaviour of the global likelihood function was restricted to $|M_1|\lesssim 1 \,\mathrm {TeV}$ and ${m_{\tilde{\ell }}}\lesssim 1 \,\mathrm {TeV}$. In order to achieve high resolution efficiently, we restricted the range of ${m_{\tilde{\ell }}}$ to $<2 \,\mathrm {TeV}$ in the full scan.^5^ To study properly the impact of the $(g-2)_{\mu }$, we performed separate sampling campaigns with and without it. On the other hand, during the sampling phase the constraints coming from LHC13 results have not been included. Since their impact consists in providing lower bounds to the sparticle masses, this choice allows for a proper assessment of their impact on the full parameter space. Moreover, we also performed dedicated scans for various DM annihilation mechanisms, in such a way to improve the quality of the sample in the description of the fine-tuned spectrum configurations that characterize them. The data sets from the various campaigns have been merged into a single set on which the likelihood is computed dynamically including or excluding the $(g-2)_\mu$ and/or the LHC13 constraints according to our interest. The total number of points in our pMSSM11 parameter scan is $\sim 2 \times 10^{9}$.

### MasterCode

We perform a global likelihood analysis of the pMSSM11 including constraints from direct searches for SUSY particles at the LHC, measurements of the Higgs boson mass and signal strengths, LHC searches for SUSY Higgs bosons, precision electroweak observables, flavour constraints from *B*- and *K*-physics observables, the cosmological constraint on the overall cold dark matter (CDM) density, and upper limits on spin-independent and -dependent LSP-nuclear scattering. We treat $(g-2)_\mu$ as an optional constraint, presenting results from global fits with and without it, and we treat $m_t$, $\alpha _s$ and $M_Z$ as nuisance parameters.

The observables contributing to the likelihood are calculated using the MasterCode tool [7–13, 33, 34, 51, 60], which interfaces and combines consistently various public and private codes using the SUSY Les Houches Accord (SLHA) [61]. The following codes are used in this analysis: SoftSusy 3.3.9 [62] for the spectrum, FeynWZ [63, 64] for the electroweak precision observables,^6^ FeynHiggs 2.11.3 [66–71] for the Higgs sector^7^ and $(g-2)_\mu$, SuFla [79, 80] and SuperIso [81–83] for the flavour physics observables, Micromegas-3.2 [84] for the DM relic density, SSARD [85] for the spin-independent and -dependent elastic scattering cross-sections $\sigma ^\mathrm{SI}_p$ and $\sigma ^\mathrm{SD}_p$,^8^ SDECAY 1.3b [89] for calculating sparticle branching ratios, and HiggsSignals 1.4.0 [90, 91] and HiggsBounds 4.3.1 [92–95] for calculating constraints on the SUSY Higgs sector.

### Electroweak and flavour constraints

Our treatments of many of these constraints follow those we have used previously, which were summarized most recently in Table 1 in [34]. Table 2 summarizes the updates we make in this paper. As noted there, the only change in the electroweak sector is in $M_W$.^9^ Here we follow [96] in combining naively the recent ATLAS measurement $M_W= 80.370 \pm 0.019 \,\mathrm {GeV}$ with the previous world average value $M_W= 80.385 \pm 0.015 \,\mathrm {GeV}$, obtaining $M_W= 80.379 \pm 0.012 \,\mathrm {GeV}$.^10^

Since one of our objectives in this paper is to emphasize the impact on the pMSSM11 parameter space of the $(g-2)_\mu$ constraint, for reference we also include in Table 2 the implementation of this constraint that we use as an option.^11^

**Table 2:** Experimental constraints that we update in this work compared to Table 1 in [34]. We indicate separately the experimental and applicable theoretical errors in the SM and SUSY (sometimes in combination, labelled “MSSM”). The contribution of the $\tau (B_s \rightarrow \mu ^+ \mu ^-)$ constraint to the global $\chi ^2$ likelihood function is essentially constant across the relevant region of the pMSSM11 parameter space, and it is not included in the fit. The new LHC constraints are all based on $\sim 36$/fb of data at 13 TeV

Observable	Source Th./Ex.	Constraint
$M_W$ [GeV]	[63, 64]/[98, 99]	$80.379 \pm 0.012\pm 0.010_\mathrm{{MSSM}}$
$a_{\mu }^\mathrm{EXP} - a_{\mu }^\mathrm{SM}$	[100–107]/[108, 109]	$(30.2 \pm 8.8 \pm 2.0_\mathrm{{MSSM}})\times 10^{-10}$
${R_{{\mu \mu }}}$	[110–112]	2D likelihood, MFV
$\tau (B_s \rightarrow \mu ^+ \mu ^-)$	[112]	$2.04 \pm 0.44 (\mathrm{stat.}) \pm 0.05 (\mathrm{syst.})$ ps
$\hbox {BR}{_{{b \rightarrow s \gamma }}^{\mathrm{EXP/SM}}}$	[113]/[114]	${0.988 \pm 0.045_{\mathrm{EXP}} \pm 0.068_{\mathrm{TH, SM}} \pm 0.050_{\mathrm{TH,SUSY}}}$
$\hbox {BR}{_{B \rightarrow \tau \nu }^{\mathrm{EXP/SM}}}$	[114, 115]	${0.883 \pm 0.158_{\mathrm{EXP}} \pm 0.096_{\mathrm{SM}}}$
${BR}_{B \rightarrow X_s \ell \ell }^\mathrm{EXP/SM}$	[116]/[114]	${0.966 \pm 0.278_{\mathrm{EXP}} \pm 0.037_{\mathrm{SM}}}$
${\Delta M}_{B_s}^{\mathrm{EXP/SM}}$	[79, 80, 117]/[114]	${0.968 \pm 0.001_{\mathrm{EXP}} \pm 0.078_{\mathrm{SM}}}$
${\frac{{\Delta M}_{B_s}^{\mathrm{EXP/SM}}}{{\Delta M}_{B_d}^{\mathrm{EXP/SM}}}}$	[79, 80, 117]/[114]	${1.007 \pm 0.004_{\mathrm{EXP}} \pm 0.116_{\mathrm{SM}}}$
${BR}_{K \rightarrow \mu \nu }^{\mathrm{EXP/SM}}$	[79, 80, 118]/[119]	${1.0005 \pm 0.0017_{\mathrm{EXP}} \pm 0.0093_{\mathrm{TH}}}$
${BR}_{K \rightarrow \pi \nu \bar{\nu }}^{\mathrm{EXP/SM}}$	[120]/[121]	${2.01 \pm 1.30_{\mathrm{EXP}} \pm 0.18_{\mathrm{SM}}}$
$\sigma ^\mathrm{SI}_p$	[3, 4, 6]	Combined likelihood in the $(m_{\tilde{\chi }^0_{1}}, \sigma ^\mathrm{SI}_p)$ plane
$\sigma ^\mathrm{SD}_p$	[5]	Likelihood in the $(m_{\tilde{\chi }^0_{1}}, \sigma ^\mathrm{SD}_p)$ plane
${\tilde{g}} \rightarrow q \bar{q} \tilde{\chi }^0_{1}, b \bar{b} \tilde{\chi }^0_{1}, t \bar{t} \tilde{\chi }^0_{1}$	[14, 15]	Combined likelihood in the ${(m_{\tilde{g}}, m_{\tilde{\chi }_1^0})}$ plane
${\tilde{q}\rightarrow q \tilde{\chi }^0_{1}}$	[14]	Likelihood in the $(m_{\tilde{q}}, m_{\tilde{\chi }^0_{1}})$ plane
${\tilde{b}} \rightarrow b \tilde{\chi }^0_{1}$	[14]	Likelihood in the ${(m_{\tilde{b}}, m_{\tilde{\chi }_1^0})}$, plane
${\tilde{t}_1} \rightarrow t \tilde{\chi }^0_{1}, c \tilde{\chi }^0_{1}, b \tilde{\chi }^\pm _{1}$	[14]	Likelihood in the ${(m_{\tilde{t}_1}, m_{\tilde{\chi }_1^0})}$, plane
$\tilde{\chi }^\pm _{1} \rightarrow \nu \ell ^\pm \tilde{\chi }^0_{1}, \nu \tau ^\pm \tilde{\chi }^0_{1}, W^\pm \tilde{\chi }^0_{1}$	[16]	Likelihood in the $(m_{\tilde{\chi }^\pm _{1}}, m_{\tilde{\chi }^0_{1}})$ plane
$\tilde{\chi }^0_{2} \rightarrow \ell ^+ \ell ^- \tilde{\chi }^0_{1}, \tau ^+ \tau ^- \tilde{\chi }^0_{1}, Z \tilde{\chi }^0_{1}$	[16]	Likelihood in the $(m_{\tilde{\chi }^0_{2}}, m_{\tilde{\chi }^0_{1}})$ plane
Heavy stable charged particles	[122]	Fast simulation based on [122, 123]
${H/A \rightarrow \tau ^+ \tau ^-}$	[124–127]	Likelihood in the $(M_A, \tan \beta )$ plane

As can be seen in Table 2, we have also updated a number of flavour constraints. In particular, we have updated the global analysis of $\mathrm{BR}(B_{s, d} \rightarrow \mu ^+\mu ^-)$ to include the latest Run 2 result from LHCb [112] as well as the Run 1 results of CMS, LHCb [110] and ATLAS [111]. We assume minimal flavour violation (MFV) when combining the $\mathrm{BR}(B_d \rightarrow \mu ^+\mu ^-)$ constraint with that from $\mathrm{BR}(B_s \rightarrow \mu ^+\mu ^-)$ into the quantity $R_{\mu \mu }$ [11], and take into account the correlation between the theoretical calculations of $f_{B_s}$ and $f_{B_d}$.

The LHCb Collaboration has also published [112] a first determination of the effective $B_s$ lifetime as measured in $B_s \rightarrow \mu ^+ \mu ^-$ decays, providing a constraint on the quantity $A_{\Delta \Gamma }$ via the relation

2 $$\begin{aligned} \frac{\tau (B_s \rightarrow \mu ^+ \mu ^-)}{\tau (B_s \rightarrow \mu ^+ \mu ^-)|_\mathrm{SM}} \; = \; \frac{1 + 2 A_{\Delta \Gamma } y_s + y_s^2}{(1 + y_s)(1+ A_{\Delta \Gamma } y_s)}, \end{aligned}$$

where [114]

3 $$\begin{aligned} y_s= & {} \tau _{B_s} \frac{ \Delta \Gamma _s}{2} \; = \; 0.0675 \pm 0.004, \nonumber \\ A_{\Delta \Gamma }\equiv & {} -2 \frac{\mathcal{R}e(\lambda )}{(1 + |\lambda |^2)}, \lambda \equiv \frac{q}{p} \frac{A(\bar{B_s} \rightarrow \mu ^+ \mu ^-)}{A({B_s} \rightarrow \mu ^+ \mu ^-)}, \end{aligned}$$

where $\tau _{B_s}$ is the inclusive $B_s$ decay lifetime, the complex numbers *p*, *q* specify the relation between the mass eigenstates of the $B_s^0 - \bar{B^0_s}$ system and the flavour eigenstates [114], and $A(B_s^0 \rightarrow \mu ^+ \mu ^-)$ and $A(\bar{B^0_s} \rightarrow \mu ^+ \mu ^-)$ are the $B_s^0$ and $\bar{B^0_s}$ decay amplitudes. In the Standard Model (SM), $A_{\Delta \Gamma }= 1$ so that $\tau (B_s \rightarrow \mu ^+ \mu ^-){|_\mathrm{SM}} = \tau _{B_s}/(1 - y_s) = 1.619 \pm 0.009$ ps. On general grounds, $A_{\Delta \Gamma } \in [-1, 1]$. The LHCb measurement $\tau (B_s \rightarrow \mu ^+ \mu ^-) = 2.04 \pm 0.44 (\mathrm{stat.}) \pm 0.05 (\mathrm{syst.})$ ps corresponds formally to $A_{\Delta \Gamma } = 7.7 \pm 10.0$, implying that the current LHCb result does not constrain significantly the pMSSM11 parameter space, and we do not include it in our fit. However, in the later discussion of our fit results we present for information the $\chi ^2$ profile likelihood functions we find for $A_{\Delta \Gamma }$ and $\tau (B_s \rightarrow \mu ^+ \mu ^-)$.

We have also updated our implementations of $b \rightarrow s \gamma$, $B \rightarrow \tau \nu$, $B \rightarrow X_s \ell \ell$, ${\Delta M}_{B_s}$ and ${\Delta M}_{B_d}$ to take account of updated theoretical calculations within the SM. For the same reason, in the kaon sector we have also updated our implementations of $K \rightarrow \mu \nu$ and $K \rightarrow \pi \nu \bar{\nu }$.^12^ Since there are, in general, supersymmetric contributions to the observables commonly used in global fits to CKM parameters, we remove these contributions and make a global fit to the CKM parameters without them.

In general, we treat the electroweak precision observables, $(g-2)_\mu$ and all *B*- and *K*-physics observables (except for $\mathrm{BR}(B_{s, d} \rightarrow \mu ^+\mu ^-)$) as Gaussian constraints, combining in quadrature the experimental and applicable SM and SUSY theory errors.

### Dark matter constraints and mechanisms

*Cosmological density*

Since we work in the framework of the MSSM, *R*-parity is conserved, so that the lightest SUSY particle (LSP) is a candidate to provide the CDM. We assume that the LSP is the lightest neutralino $\tilde{\chi }^0_{1}$ [19, 20], and that it is the dominant component of the CDM. As in our recent papers [33, 34], we use the Planck 2015 constraint on the total CDM density: $\Omega _\mathrm{CDM} h^2 = 0.1186 \pm 0.0020_\mathrm{EXP} \pm 0.0024_\mathrm{TH}$ [128].

*Density mechanisms*

As one of the primary objectives in our analysis is to investigate the relevances of various mechanisms for bringing the relic $\tilde{\chi }^0_{1}$ density into the range allowed by astrophysics and cosmology, we introduce a set of measures related to particle masses that were found in our previous analyses [51] to indicate when specific mechanisms were dominant.^13^ These may be grouped as follows.

- *Coannihilation with an Ino*
  This may be important if the $\tilde{\chi }^0_{1}$ is not much lighter than the lighter chargino, $\tilde{\chi }^\pm _{1}$, and the second neutralino, $\tilde{\chi }^0_{2}$, or the gluino, ${\tilde{g}}$. For these cases we introduce the coannihilation measures
  4 $$\begin{aligned} \text {Ino coann.}: \quad \left( \frac{M_\mathrm{Ino}}{m_{\tilde{\chi }^0_{1}}} - 1 \right) < 0.25. \end{aligned}$$
  We find that chargino and $\tilde{\chi }^0_{2}$ coannihilation is important in our analysis, and in our 2-dimensional plots we shade green the regions where (4) is satisfied when the Ino is the lighter chargino, $\tilde{\chi }^\pm _{1}$ (which is almost degenerate with the $\tilde{\chi }^0_{2}$). On the other hand, we find that gluino coannihilation is not important in the pMSSM11 when the $(g-2)_\mu$ constraint is imposed. This is due to the fact that $(g-2)_\mu$ forces the neutralino mass to values for which a gluino of equivalent mass would be excluded by current LHC results.
- *Coannihilation with sleptons*
  In the version of the pMSSM that we study here, the two stau mass eigenvalues are similar, since the soft SUSY-breaking parameters are specified at the TeV scale and the left-right mixing $\propto m_\tau$ is relatively small, but the stau masses are not degenerate with the selectron and smuon masses, in general. We find that smuon and selectron coannihilation are in general more important than stau coannihilation, thanks to the greater multiplicity of near-degenerate states. We introduce the following coannihilation measure:
  5 $$\begin{aligned} {{\tilde{\ell }}} \text { coann.}: \quad \left( \frac{m_{\tilde{\ell }}}{m_{\tilde{\chi }^0_{1}}} - 1 \right) < 0.15, \end{aligned}$$
  and shade in yellow (pink) the regions of our two-dimensional plots where (5) is satisfied for $\ell = \mu , e$ ($\tau$), respectively.
- *Coannihilation with squarks*
  Similarly, this may be important for squarks $\tilde{q}$ that are not much heavier than the $\tilde{\chi }^0_{1}$. The case considered most often has been ${\tilde{q}} = {\tilde{t}}_1$, but here we consider all possibilities, including coannihilations with first- and second-generation squarks, which we find to be important when the LHC 13-TeV constraint or $(g-2)_\mu$ is dropped. We introduce the coannihilation measure
  6 $$\begin{aligned} {\tilde{q}} \text { coann.}: \quad \left( \frac{m_{\tilde{q}}}{m_{\tilde{\chi }^0_{1}}} - 1 \right) < 0.15, \end{aligned}$$
  and we use the following colours in our plots for the regions where (6) is satisfied: ${\tilde{q}} = {\tilde{d}}/{\tilde{s}}/{\tilde{u}}/{\tilde{c}}_{L,R}$ cyan, ${\tilde{t}}_{1}$ grey, ${\tilde{b}}_1$ purple.
- *Annihilation via a direct-channel boson pole*
  When there is a massive boson *B* with mass $M_B \sim 2 m_{\tilde{\chi }^0_{1}}$, $\tilde{\chi }^0_{1} \tilde{\chi }^0_{1}$ annihilation is enhanced along a ‘funnel’ in parameter space. We have found that such a mechanism is likely to dominate if the following condition is satisfied:
  7 $$\begin{aligned} B \text { funnel:} \quad \left| \frac{M_B}{m_{\tilde{\chi }^0_{1}}} - 2 \right| <0.1. \end{aligned}$$
  We have considered the cases $B = h, Z$ and *H* / *A*, and use blue shading for the regions of our subsequent plots where (7) is satisfied when $B = H/A$. We comment later on a small region where rapid annihilation via the *h* and *Z* poles is important.
- *Enhanced Higgsino component*
  We have also considered a somewhat different possibility, namely that the $\tilde{\chi }^0_{1}$ has an enhanced Higgsino component because the following condition is satisfied, which is similar to the situation in the focus-point region of the CMSSM:
  8 $$\begin{aligned} \text {Higgsino:} \quad \left| \left( \frac{\mu }{m_{\tilde{\chi }^0_{1}}} \right) - 1 \right| <0.3. \end{aligned}$$
  Regions where the condition (8) is satisfied generally satisfy the chargino coannihilation condition with a Higgsino-like LSP, and are also shaded green.
- *Hybrid regions*
  In addition to the ‘primary’ regions where only one of the conditions (4)–(8) is satisfied, there are also ‘hybrid’ regions where more than one condition is satisfied. These are indicated in the following by mixtures of the corresponding primary colours.

*Direct DM searches*

We implement experimental constraints from direct searches for supersymmetric DM via both spin-independent and -dependent scattering on nuclei. We use the LUX [4], XENON1T [6] and PandaX-II [3] constraints on the spin-independent DM scattering cross section $\sigma ^\mathrm{SI}_p$, which we implement via a combined two-dimensional likelihood function in the $(m_{\tilde{\chi }^0_{1}}, \sigma ^\mathrm{SI}_p)$ plane.

Our treatment of the spin-independent nuclear scattering matrix element follows that in our previous work [12] and is based on SSARD [85]. As reviewed, for example, in [88] the largest uncertainties in the matrix element are those associated with the pion-nucleon $\sigma$-term, $\Sigma _{\pi N}$, and the SU(3) octet symmetry-breaking contribution to the nucleon mass, $\sigma _0$. These may be expressed as follows in terms of ${\bar{q}} q$ matrix elements in the nucleon:

9 $$\begin{aligned} \Sigma _{\pi N}= & {} \frac{m_u + m_d}{2} \langle N | {\bar{u}} u + {\bar{d}} d | N \rangle , \nonumber \\ \sigma _0= & {} \frac{m_u + m_d}{2} \langle N | {\bar{u}} u + {\bar{d}} d - 2 {\bar{s}} s | N \rangle , \end{aligned}$$

from which we see that the ${\bar{s}} s$ matrix element

10 $$\begin{aligned} y \; \equiv \; \frac{ 2 \langle N | {\bar{s}} s | N \rangle }{ \langle N | {\bar{u}} u + {\bar{d}} d | N \rangle } \; = \; 1 - \frac{\sigma _0}{\Sigma _{\pi N}}. \end{aligned}$$

It is well known that $\sigma ^\mathrm{SI}_p$ is sensitive to the value of *y*, and hence to the values of $\sigma _0$ and $\Sigma _{\pi N}$. We follow [129] in interpreting the measured octet baryon mass differences as yielding $\sigma _0 = 36 \pm 7 \,\mathrm {MeV}$,^14^ and we follow our previous work in assuming here that $\Sigma _{\pi N} = 50 \pm 7 \,\mathrm {MeV}$,^15^ corresponding to a central value of $y = 0.28$. For comparison, two recent determinations of $\Sigma _{\pi N}$ give somewhat larger values that are, however, compatible with the value we assume, within the quoted uncertainties: $\Sigma _{\pi N} = 59.1 \pm 3.5 \,\mathrm {MeV}$ (from pionic atoms) [132] and $58 \pm 5 \,\mathrm {MeV}$ (from $\pi$-nucleon scattering) [133] (see also [134], which found the value $\Sigma _{\pi N} = 59 \pm 7 \,\mathrm {MeV}$). On the other hand, lattice calculations [135–138] yield systematically smaller values of $\Sigma _{\pi N}$ that are in tension with these data-driven estimates, as discussed in [133]. Our value of $\Sigma _{\pi N}$ is intermediate and relatively conservative in that it implies a smaller value of *y* than the data-driven estimates of $\Sigma _{\pi N}$.^16^

We also implement in this paper the PICO [5] constraint on the spin-dependent DM scattering cross section $\sigma ^\mathrm{SD}_p$, also using the SSARD code [85]. As discussed in [139], the spin-dependent $\tilde{\chi }^0_{1} p$ scattering matrix element is determined by the light quark contributions to the proton spin, which we take to be [88]

11 $$\begin{aligned}&\Delta u = + 0.84 \pm 0.03, \nonumber \\&\Delta d = - 0.43 \pm 0.03, \nonumber \\&\Delta s = - 0.09 \pm 0.03, \end{aligned}$$

where the uncertainties are dominated by those in measurements of polarized deep-inelastic scattering, and hence are correlated: the uncertainty in the combination $\Delta u - \Delta d$ (from $g_A$) is very small, and that in $\Delta u + \Delta d - 2 \Delta s$ (from semileptonic octet baryon decays) is also somewhat smaller.^17^

*Indirect astrophysical searches for DM*

These include searches for $\gamma$-rays from DM annihilations near the Galactic centre and in dwarf galaxies, and for energetic neutrinos produced by the annihilations of DM particles trapped inside the Sun. There are large astrophysical uncertainties in estimates of the possible $\gamma$-ray flux from the Galactic centre, and other studies have indicated that the available limits on the fluxes from dwarf galaxies do not yet impose competitive constraints on supersymmetric models - see, for example, [140] and [32]. The strongest constraints on energetic solar neutrinos are those provided by the IceCube Collaboration [141]. Their impact depends on the annihilation final states, being strongest for annihilations into $\tau ^+ \tau ^-$, somewhat weaker for $W^+ W^-$, and much weaker for ${\bar{b}} b$ final states.

The capture of dark matter particles in the Sun is often assumed to be dominated by energy loss due to spin-dependent scattering on protons, in which case an upper limit on the neutrino flux may be used to constrain the spin-dependent cross-section $\sigma ^\mathrm{SD}_p$, as done by the IceCube Collaboration [141]. However, the interpretation of this constraint [141] depends on the importance of spin-independent scattering on $^4$He and heavier nuclei inside the Sun, and whether the DM density inside the Sun is in equilibrium between capture and annihilation [142]. As discussed in Sect. 4.10, we have found in an exploratory study that the IceCube constraint has little impact once the more recent PICO constraint [5] on $\sigma ^\mathrm{SD}_p$ is taken into account. In view of the fact that it has fewer uncertainties, we use the PICO result in our global fit, setting aside the IceCube result [141].^18^

### 13 TeV LHC constraints

The LHC constraints we consider are those from searches for coloured sparticles in events with missing transverse energy, $/ E_T$, accompanied by jets and possibly leptons, searches for electroweak inos in events with multiple leptons, searches for long-lived charged particles, measurements of the 125 GeV Higgs boson *h*, and searches for the heavier SUSY Higgs bosons $H, A, H^\pm$. Our principal focus in this paper is on the implications of Run-2 LHC searches with $\sim 36$/fb of data at 13 TeV, though we also make comparisons with the situation before these constraints were released. Our implementations of the constraints from LHC Run 1 at energies of 7 and 8 TeV used in our previous analysis of the pMSSM10 model were described in [13], and our implementations of $/ E_T$ searches with $\sim 13$/fb of data at 13 TeV in the gluino and squark production channels were described in [34], as were our implementations of searches for long-lived charged particles and for $H, A, H^\pm$ with similar data sets. We refer the reader to these publications for details of those implementations, focusing here on our implementations of the Run 2 searches with $\sim 36$/fb of data.

*Searches for gluinos and squarks*

We consider the constraints from CMS simplified model searches using events with $/ E_T$ and jets but no leptons released in [14] and events with $/ E_T$ and jets and a single lepton released in [15].

In the approach taken, e.g., by CheckMATE [143], ColliderBit [144] and MadAnalysis 5 [145], Monte Carlo simulations are used to estimate the signal yield from a model point after the event selection and to test it by comparing it with the upper bound given by an experimental collaboration. However, such a method is time-consuming and computationally prohibitive for our purpose. To circumvent this issue, we take the Fastlim [146] approach^19^ and consider the implications of [14] for the following supersymmetric topologies: ${\tilde{g}}{\tilde{g}}\rightarrow [ q {\bar{q}} \tilde{\chi }^0_{1} ]^2$ and $[ b {\bar{b}} \tilde{\chi }^0_{1} ]^2$, and ${\tilde{q}} {\tilde{\bar{q}}} \rightarrow [ q \tilde{\chi }^0_{1} ] [{\bar{q}} \tilde{\chi }^0_{1} ]$, and the implications of [15] for the topology ${\tilde{g}} {\tilde{g}} \rightarrow [ t {\bar{t}} \tilde{\chi }^0_{1} ]^2$. The kinematics of each of these topologies depends on a reduced subset of sparticle masses, e.g., $(m_{\tilde{g}}, m_{\tilde{\chi }^0_{1}})$ in the case of the ${\tilde{g}}{\tilde{g}}\rightarrow [ q {\bar{q}} \tilde{\chi }^0_{1} ]^2$ topology, and the CMS publications [14, 15] provide in Root files 95% CL upper limits $\sigma _\mathrm{UL}$ on the cross sections in the corresponding parameter planes. For each point in the main pMSSM11 sample, we calculate for the ${\tilde{g}}{\tilde{g}}$ initial state and various final states contributions to the global $\chi ^2$ likelihood function of the form

12 $$\begin{aligned} \chi ^2_{{\tilde{g}}\rightarrow \mathrm{SM} \tilde{\chi }^0_{1}} = 5.99 \cdot \left[ \frac{\sigma _{{\tilde{g}}{\tilde{g}}}\;\mathrm{BR}^2_{{\tilde{g}}\rightarrow \mathrm{SM} \tilde{\chi }^0_{1}}}{{\sigma _\mathrm{UL}^{{\tilde{g}}\rightarrow \mathrm{SM} \tilde{\chi }^0_{1}}}(m_{\tilde{g}}, m_{\tilde{\chi }^0_{1}})} \right] ^2, \end{aligned}$$

where SM denotes the Standard Model particles considered in each topology, $\mathrm{SM} \equiv q {\bar{q}}, b {\bar{b}}$ and $t {\bar{t}}$, and analogously for the ${\tilde{q}} {\tilde{\bar{q}}} \rightarrow [ q \tilde{\chi }^0_{1} ] [{\bar{q}} \tilde{\chi }^0_{1} ]$ topology, where $\mathrm{SM} \equiv q$ and ${\bar{q}}$. We use NLL-fast [149, 150] to compute the cross sections for coloured sparticle pair-production up to NLO+NLL level.

If gluino and squarks have comparable masses, associated gluino-squark production may be sizeable. In the $m_{{\tilde{g}}} \gtrsim m_{\tilde{q}}$ region, a fraction of the $gq \rightarrow {\tilde{g}}\tilde{q}$ process where the gluino decays into $\bar{q} + \tilde{q}$ may be regarded as the production of a squark–antisquark pair with a soft quark jet. Ignoring this soft jet, we can constrain this process by considering the $q {\bar{q}} \rightarrow \tilde{q} {\tilde{\bar{q}}}$ simplified model limit. In the analyses we consider, jets are treated inclusively and this extra quark jet tends to slightly increase the acceptance. Ignoring the soft jet therefore results in underestimation of the signal acceptance, leading to a conservative limit. In order to constrain the $g q \rightarrow {\tilde{g}}\tilde{q} \rightarrow \tilde{q} {\tilde{\bar{q}}} q$ process in the same way as $q {\bar{q}} \rightarrow \tilde{q} {\tilde{\bar{q}}}$, we rescale the squark cross-section as $\sigma _{\tilde{q} \tilde{q}} \rightarrow \sigma _{\tilde{q} \tilde{q}} + \sigma _{\tilde{g} \tilde{q}} \cdot \mathrm{BR}_{\tilde{g} \rightarrow q \tilde{q}}$ before applying squark simplified model limit.

Similarly, in the $m_{\tilde{q}} \gtrsim m_{{\tilde{g}}}$ region we rescale the gluino cross-section as $\sigma _{\tilde{g} \tilde{g}} \rightarrow \sigma _{\tilde{g} \tilde{g}} + \sigma _{\tilde{g} \tilde{q}} \cdot \mathrm{BR}_{\tilde{q} \rightarrow q \tilde{g}}$ to constrain the $g q \rightarrow {\tilde{g}}\tilde{q} \rightarrow \tilde{g} \tilde{g} q$ process using the gluino simplified model limit.

*Stop and sbottom searches*

Our treatment of LHC 13 TeV limits on stops and sbottoms is similar in principle to our implementation of the gluino and squark constraints described above. It is based on CMS simplified model searches in the jets + 0 [14] and 1 [15] lepton final states, where the results are interpreted as limits on the following topologies: ${\tilde{t}_1} {\tilde{\bar{t}}}_1 \rightarrow [ t \tilde{\chi }^0_{1} ] [{\bar{t}} \tilde{\chi }^0_{1} ]$, $[ c \tilde{\chi }^0_{1} ] [{\bar{c}} \tilde{\chi }^0_{1} ]$ in the compressed-spectrum region, $[ b W^{+} \tilde{\chi }^0_{1} ] [{\bar{b}} W^{-} \tilde{\chi }^0_{1} ]$ via $\tilde{\chi }^\pm _{1}$ intermediate states and ${\tilde{b}_1} {\tilde{\bar{b}}}_1 \rightarrow [ b \tilde{\chi }^0_{1} ] [{\bar{b}} \tilde{\chi }^0_{1} ]$. We also use Fastlim to implement the CMS constraints in all these channels, following the same procedure as described above for gluinos and squarks, and estimating the corresponding contributions to the global $\chi ^2$ likelihood function as

13 $$\begin{aligned} \chi ^2_{\tilde{q}_3 \rightarrow \mathrm{SM} \tilde{\chi }^0_{1}} = 5.99 \cdot \left[ \frac{\sigma _{\tilde{q}_3 {\tilde{\bar{q}}}_3}\;\mathrm{BR}^2_{\tilde{q}_3 \rightarrow \mathrm{SM} \tilde{\chi }^0_{1}}}{{\sigma _\mathrm{UL}^{\tilde{q}_3 \rightarrow \mathrm{SM} \tilde{\chi }^0_{1}}}(m_{\tilde{t}_{1}}, m_{\tilde{\chi }^0_{1}})} \right] ^2, \end{aligned}$$

where $\mathrm{SM} = t, c$ and $b W^{+}$ for $\tilde{q}_3 = \tilde{t}_1$ and $\mathrm{SM} = b$ for $\tilde{q}_3 = \tilde{b}_1$, respectively.

In a significant part of the pMSSM11 parameter space, the neutralino relic abundance is brought into the observed range by Wino or Higgsino coannihilation mechanisms. In these regions, $\tilde{\chi }^\pm _{1}$ and $\tilde{\chi }^0_{1}$ are highly mass degenerate, with a mass difference that is typically smaller than 5 GeV. Since the decay products of the $\tilde{\chi }^\pm _{1} \rightarrow \tilde{\chi }^0_{1}$ transition are too soft to affect the signal acceptance, we can replace $\tilde{\chi }^\pm _{1}$ by $\tilde{\chi }^0_{1}$ in the simplified topology. This approximation allows us to constrain the $\tilde{t}_1 \rightarrow b \tilde{\chi }^+_{1}$ ($\tilde{b}_1 \rightarrow t \tilde{\chi }^-_{1}$) topology using the $\tilde{b}_1 \rightarrow b \tilde{\chi }^0_{1}$ ($\tilde{t}_1 \rightarrow t \tilde{\chi }^0_{1}$) simplified model limit. Thus, in the Wino and Higgsino coannihilation regions, we replace, e.g., the numerator in (13) by $\sigma _{\tilde{t}_1 {\tilde{\bar{t}}}_1} \mathrm{BR}^2_{\tilde{t}_1 \rightarrow t \tilde{\chi }^0_{1}} \rightarrow \sigma _{\tilde{t}_1 {\tilde{\bar{t}}}_1} \mathrm{BR}^2_{\tilde{t}_1 \rightarrow t \tilde{\chi }^0_{1}} + \sigma _{\tilde{b}_1 {\tilde{\bar{b}}}_1} \mathrm{BR}^2_{\tilde{b}_1 \rightarrow t \tilde{\chi }^-_{1}}$, enhancing the sensitivity.

**Table 3:** Summary of the simplified model limits from $\sim 36$/fb of CMS data at 13 TeV used in our study

Topology	Analysis	Refs.
${\tilde{g}}{\tilde{g}}\rightarrow [\, q {\bar{q}} \tilde{\chi }^0_{1} \,]^2, \, [\, b {\bar{b}} \tilde{\chi }^0_{1} \,]^2$	0 leptons + jets with $/ E_T$	[14]
${\tilde{g}}{\tilde{g}}\rightarrow [\, t {\bar{t}} \tilde{\chi }^0_{1} \,]^2$	1 lepton + jets with $/ E_T$	[15]
${\tilde{q}} {\tilde{\bar{q}}} \rightarrow [\, q \tilde{\chi }^0_{1} \,] [\, {\bar{q}} \tilde{\chi }^0_{1} \,]$	0 leptons + jets with $/ E_T$	[14]
${\tilde{b}} {\tilde{\bar{b}}} \rightarrow [\, b \tilde{\chi }^0_{1} \,] [\, {\bar{b}} \tilde{\chi }^0_{1} \,]$	0 leptons + jets with $/ E_T$	[14]
${\tilde{t}_1} {\tilde{\bar{t}}}_1 \rightarrow [\, t \tilde{\chi }^0_{1} \,] [\, {\bar{t}} \tilde{\chi }^0_{1} \,]$, $[\, c \tilde{\chi }^0_{1} \,] [\, {\bar{c}} \tilde{\chi }^0_{1} \,]$	0 leptons + jets with $/ E_T$	[14]
${\tilde{t}_1} {\tilde{\bar{t}}}_1 \rightarrow [\, \bar{b} {\tilde{\chi }^+_{1}} \,] [\, \bar{b} {\tilde{\chi }^-_{1}} \,] \rightarrow [\, \bar{b} W^+ \tilde{\chi }^0_{1} \,] [\, \bar{b} W^- \tilde{\chi }^0_{1} \,]$	0 leptons + jets with $/ E_T$	[14]
$\tilde{\chi }^\pm _{1} \tilde{\chi }^0_{2} \rightarrow [\, \nu \ell ^\pm \tilde{\chi }^0_{1} \,] [\, \ell ^+ \ell ^- \tilde{\chi }^0_{1} \,] ~(\mathrm{via}~ \tilde{\ell }^\pm )$	Multileptons with $/ E_T$	[16]
$\tilde{\chi }^\pm _{1} \tilde{\chi }^0_{2} \rightarrow [\, \nu \tau ^\pm \tilde{\chi }^0_{1} \,] [\, \tau ^+ \tau ^- \tilde{\chi }^0_{1} \,] ~(\mathrm{via}~ \tilde{\tau }^\pm )$	Multileptons with $/ E_T$	[16]
$\tilde{\chi }^\pm _{1} \tilde{\chi }^0_{2} \rightarrow [\, W^\pm \tilde{\chi }^0_{1} \,] [\, Z \tilde{\chi }^0_{1} \,]$	Multileptons with $/ E_T$	[16]

*Searches for electroweak inos*

The CMS Collaboration has also released results from searches for electroweak ino production at the LHC in multilepton final states with $\sim 36$/fb of data at 13 TeV [16]. The signatures we have implemented are $\tilde{\chi }^\pm _{1} \tilde{\chi }^0_{2} \rightarrow [W \tilde{\chi }^0_{1}] [Z \tilde{\chi }^0_{1}], 3 \ell ^\pm + 2 \tilde{\chi }^0_{1}$ via ${\tilde{\ell }}^\pm /\tilde{\nu }$ intermediate states, and $3 \tau ^\pm + 2 \tilde{\chi }^0_{1}$ via ${\tilde{\tau }}^\pm$ intermediate states. As in the cases of searches for strongly-interacting sparticles described above, we use Fastlim to compare the cross-section times branching ratio with the 95% CL upper limit released by CMS [16]. We obtain the corresponding contributions to the global $\chi ^2$ likelihood function as

14 $$\begin{aligned}&\chi ^2_{\tilde{\chi }^\pm _{1} \rightarrow \mathrm{SM} \tilde{\chi }^0_{1}, \tilde{\chi }^0_{2} \rightarrow \mathrm{SM} \tilde{\chi }^0_{1}} \nonumber \\&\quad \simeq 5.99 \cdot \left[ \frac{\sigma _{\tilde{\chi }^\pm _{1} \tilde{\chi }^0_{2}} \mathrm{BR}_{\tilde{\chi }^\pm _{1} \rightarrow \mathrm{SM} \tilde{\chi }^0_{1}} \mathrm{BR}_{\tilde{\chi }^0_{2} \rightarrow \mathrm{SM} \tilde{\chi }^0_{1}}}{\sigma _\mathrm{UL}^{(\tilde{\chi }^\pm _{1} \rightarrow \mathrm{SM} \tilde{\chi }^0_{1})(\tilde{\chi }^0_{2} \rightarrow \mathrm{SM} \tilde{\chi }^0_{1}) }} \right] ^2, \end{aligned}$$

where $\mathrm{SM} \equiv W$ or *Z*, one or two $\ell ^\pm$ and one or two $\tau ^\pm$, respectively. One complication compared to the previous coloured sparticle cases is that $\sigma _{\tilde{\chi }^\pm _{1} \tilde{\chi }^0_{2}}$ depends on many MSSM parameters:

15 $$\begin{aligned}&\sigma (pp \rightarrow \tilde{\chi }^\pm _{1} \tilde{\chi }^0_{2})\nonumber \\&\quad = F \left( M_1, M_2, \mu , \tan \beta , m_{\tilde{q}_L}, m_{\tilde{u}_R}, m_{\tilde{d}_R} \right) , \end{aligned}$$

and it is not feasible to tabulate the cross section directly in a multi-dimensional look-up table. We have therefore used the code EWK-fast [151], which is based on the observation that $\sigma (pp \rightarrow \tilde{\chi }^\pm _{1} \tilde{\chi }^0_{2})$ factorizes mathematically (where $\tilde{\chi }_{i}$ and $\tilde{\chi }_{j}$ represent any chargino and/or neutralino):

16 $$\begin{aligned} \sigma (pp \rightarrow {\tilde{\chi }_i} {\tilde{\chi }_j}) \; = \; \sum _a T_a (\mathcal{U}) F_a \left( m_{\tilde{\chi }_i}, m_{\tilde{\chi }_j}, m_a \right) , \end{aligned}$$

where $T_a (\mathcal{U})$ is a function of the mixing matrices $\mathcal{U} = \{U, V, N\}$ that can be calculated analytically. The factor $F_a(m_{\tilde{\chi }_i}, m_{\tilde{\chi }_j}, m_a)$ captures the kinematics and the effect of the parton distribution function and is tabulated in 3-dimensional look-up tables as a function of $m_{\tilde{\chi }_i}, m_{\tilde{\chi }_j}$ and $m_a$, where $m_a = m_{\tilde{q}_L}, m_{\tilde{u}_R}$ or $m_{\tilde{d}_R}$.

The electroweak ino analyses described above can be extended to constrain models in which electroweak inos can be produced in the decays of coloured sparticles. This is because these searches do not impose conditions on the number of jets and the final states in such events resemble those arising from the direct production of electroweak inos associated with initial-state QCD radiation. In order to constrain this class of events we include an extra contribution to the electroweak ino cross-section, much as we discussed above in the case of the $\tilde{q} \tilde{g}$ constraint. For example, in order to constrain $\tilde{q} {\tilde{\bar{q}}} \rightarrow \tilde{\chi }_i \tilde{\chi }_j + \mathrm{jets}$, we rescale the cross-section: $\sigma _{{\tilde{\chi }_i} {\tilde{\chi }_j}} \rightarrow \sigma _{{\tilde{\chi }_i} {\tilde{\chi }_j}} + \sigma _{\tilde{q} {\tilde{\bar{q}}}} \, \mathrm{BR}_{\tilde{q} \rightarrow j \tilde{\chi }_i} \, \mathrm{BR}_{{\tilde{\bar{q}}} \rightarrow j \tilde{\chi }_j}$ before applying the electroweak ino simplified limit.^20^

### Combination of contributions to global ${\varvec{\chi }}^\mathbf{2}$ function from LHC sparticle searches

The total contribution of LHC Run-2 sparticle searches is obtained by adding the contributions from the coloured sparticle (12) and (13) and electroweak ino searches (14):

17 $$\begin{aligned} \chi ^2_\mathrm{LHC~Run~2} \; = \; \sum _i^\mathrm{Topologies} \chi ^2_i, \end{aligned}$$

where the sum is over all the distinct SM final states mentioned above. The simple sum is justified because event samples with different final states are statistically independent, so that their correlations are not important for our analysis. We summarise the simplified model limits we use in our scan in Table 3.

### Measurements of the ${\varvec{h}}{} \mathbf{(125)}$ boson

These are incorporated via the HiggsSignals code [90, 91], which implements the information from ATLAS and CMS measurements from LHC Run 1, as summarized in the joint ATLAS and CMS publication [152].

### Searches for heavy MSSM Higgs bosons

These are incorporated via the HiggsBounds code [92–95], which implements the information from ATLAS and CMS measurements from LHC Run 1, supplemented by the constraint from $\sim 36$/fb of data from the LHC at 13 TeV provided by ATLAS [127].

### Searches for long-lived or stable charged particles

The CMS Collaboration has published a search for charged particles with lifetimes $\gtrsim 3$ ns [123], and a search for massive charged particles that leave the detector without decaying [122]. We do not include the results of these searches in our global likelihood analysis, but comment later on their potential impacts. The only constraint that we impose on long-lived charged sparticles *a priori* is to require the lifetime to be smaller than $10^3$ s so as to avoid modifying the successful predictions of cosmological nucleosynthesis calculations [153–159].

**Table 4:** Values of the pMSSM11 input parameters and values of the global $\chi ^2$ function at the best-fit points including the LHC 13-TeV constraints, with and without the $(g-2)_\mu$ constraint, as well as at representative points in the ‘nose’ regions in the top left and right panels of Fig. 2. Lower rows show the total $\chi ^2/$d.o.f. and the corresponding p-values for each point. As discussed in the text, we calculate these omitting the contributions from HiggsSignals, which are shown separately in the last line. The SLHA files for these points are available on our website, at the following URL https://mastercode.web.cern.ch/mastercode/downloads.php

Parameter	With LHC 13 TeV and $(g-2)_\mu$		With LHC 13 TeV, not $(g-2)_\mu$	
	Best fit	‘Nose’ region	Best fit	‘Nose’ region
$M_1$	0.25$\,\mathrm {TeV}$	$-$ 0.39$\,\mathrm {TeV}$	$-$ 1.3$\,\mathrm {TeV}$	$-$ 1.5$\,\mathrm {TeV}$
$M_2$	0.25$\,\mathrm {TeV}$	1.2$\,\mathrm {TeV}$	2.3$\,\mathrm {TeV}$	2.0$\,\mathrm {TeV}$
$M_3$	$-$ 3.86$\,\mathrm {TeV}$	$-$ 1.7$\,\mathrm {TeV}$	1.9$\,\mathrm {TeV}$	1.0$\,\mathrm {TeV}$
$m_{\tilde{q}}$	4.0$\,\mathrm {TeV}$	2.00$\,\mathrm {TeV}$	0.9$\,\mathrm {TeV}$	0.9$\,\mathrm {TeV}$
$m_{\tilde{q}_3}$	1.7$\,\mathrm {TeV}$	4.1$\,\mathrm {TeV}$	2.0$\,\mathrm {TeV}$	1.9$\,\mathrm {TeV}$
$m_{\tilde{\ell }}$	0.35$\,\mathrm {TeV}$	0.36$\,\mathrm {TeV}$	1.9$\,\mathrm {TeV}$	1.4$\,\mathrm {TeV}$
$m_{\tilde{\tau }}$	0.46$\,\mathrm {TeV}$	1.4$\,\mathrm {TeV}$	1.3$\,\mathrm {TeV}$	1.4$\,\mathrm {TeV}$
$M_A$	4.0$\,\mathrm {TeV}$	4.2$\,\mathrm {TeV}$	3.0$\,\mathrm {TeV}$	3.3$\,\mathrm {TeV}$
*A*	2.8$\,\mathrm {TeV}$	5.4$\,\mathrm {TeV}$	$-$ 3.4$\,\mathrm {TeV}$	$-$ 3.4$\,\mathrm {TeV}$
$\mu$	1.33$\,\mathrm {TeV}$	$-$ 5.7$\,\mathrm {TeV}$	$-$ 0.95$\,\mathrm {TeV}$	$-$ 0.93$\,\mathrm {TeV}$
$\tan \beta$	36	19	33	33
$\chi ^2/\hbox {d.o.f.}$	22.1/20	24.46/20	20.88/19	22.57/19
p-value	0.33	0.22	0.34	0.25
$\chi ^2(HS)$	68.01	67.97	68.06	68.05

**Fig. 1:**
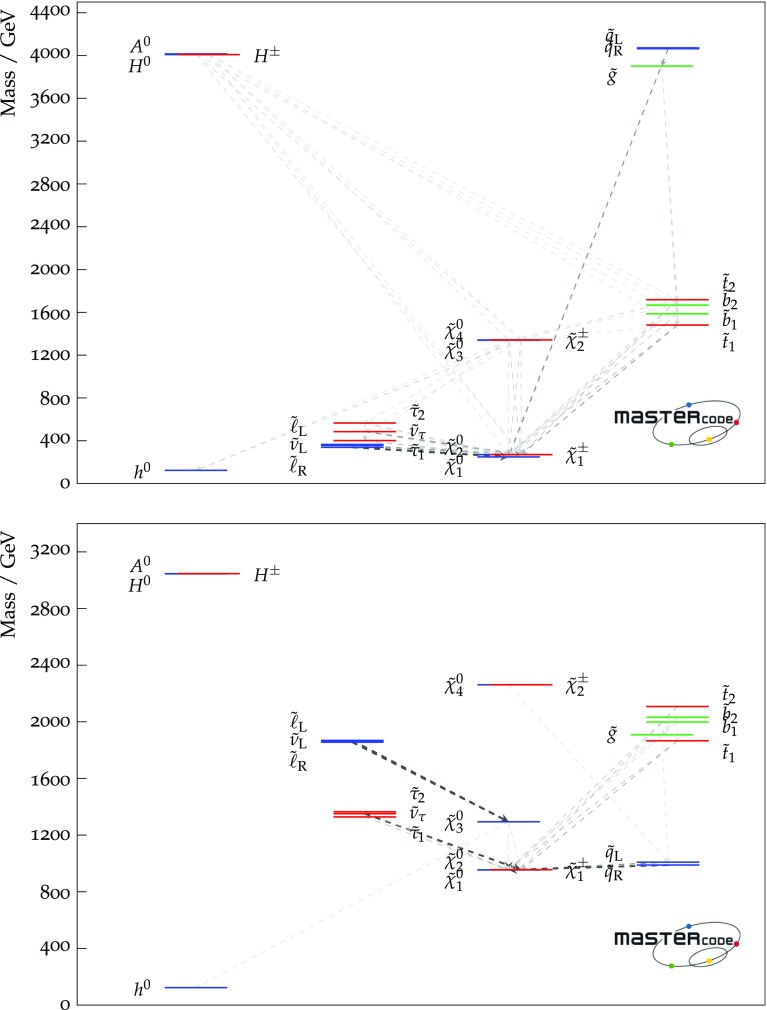
Higgs and sparticle spectra for the best-fit points for the pMSSM11 with (top) and without the $(g-2)_\mu$ constraint (bottom), showing also decay paths with branching ratios $> 5\%$, the widths of the lines being proportional to the branching ratios. These plots were prepared using the code presented in [160]

## Global fit results

The input parameter values for our best-fit points with and without $(g-2)_\mu$ are shown in the second and fourth columns of Table 4, and the spectra and dominant decays shown in Fig. 1. The third and fifth columns show input values for other points of interest that we discuss below. Lower rows of Table 4 show the total $\chi ^2$ per degree of freedom (d.o.f.) for each point, dropping the contributions from HiggsSignals that are shown in the last line. We also show the corresponding p-values, as calculated using the prescription described in [34] to estimate the number of degrees of freedom.^21^ We ignored the contribution to the likelihood coming from the nuisance parameters, and we removed the contribution to the likelihood from HiggsSignals, so as to avoid biasing our results by giving too much importance to the Higgs signal rates. Since all the other constraints contribute significantly to $\chi ^2$ function somewhere in the pMSSM11, we include them all in the d.o.f. count. However, we merged into a single constraint the LHC direct searches for sparticle production at 8 and 13 TeV, and also combined the 8- and 13-TeV limits on heavy Higgs bosons from $A/H \rightarrow \tau ^+ \tau ^-$ searches. This results in totals of 31 and 30 constraints for the cases with and without $(g-2)_\mu$, respectively. Since the number of free parameters is 11, this yields 20 and 19 for the numbers of d.o.f. in the two cases, as stated in Table 4. We note that the p-values are all comfortably high, whether $(g-2)_\mu$ is included, or not.

### Parameter planes

We now display results from our global fits with and without $(g-2)_\mu$ in pairs of 2-dimensional pMSSM11 parameter planes. We indicate the locations of the best-fit points in these two-dimensional projections by green stars, We also show in these planes the $\Delta \chi ^2 = 2.30, 5.99$ and 11.3 contours, corresponding approximately to the boundaries of the regions preferred/allowed/possible at the 1-/2-/3-$\sigma$ levels (68%, 95% and 99.7% CL), as red, blue and green solid lines, respectively. Within the 2-$\sigma$ contours, we use colour coding to indicate the dominant DM mechanisms, as discussed in Sect. 2.4, for the parameter sets that minimize $\chi ^2$ at each point in the plane.

*Squarks and gluinos*

**Fig. 2:**
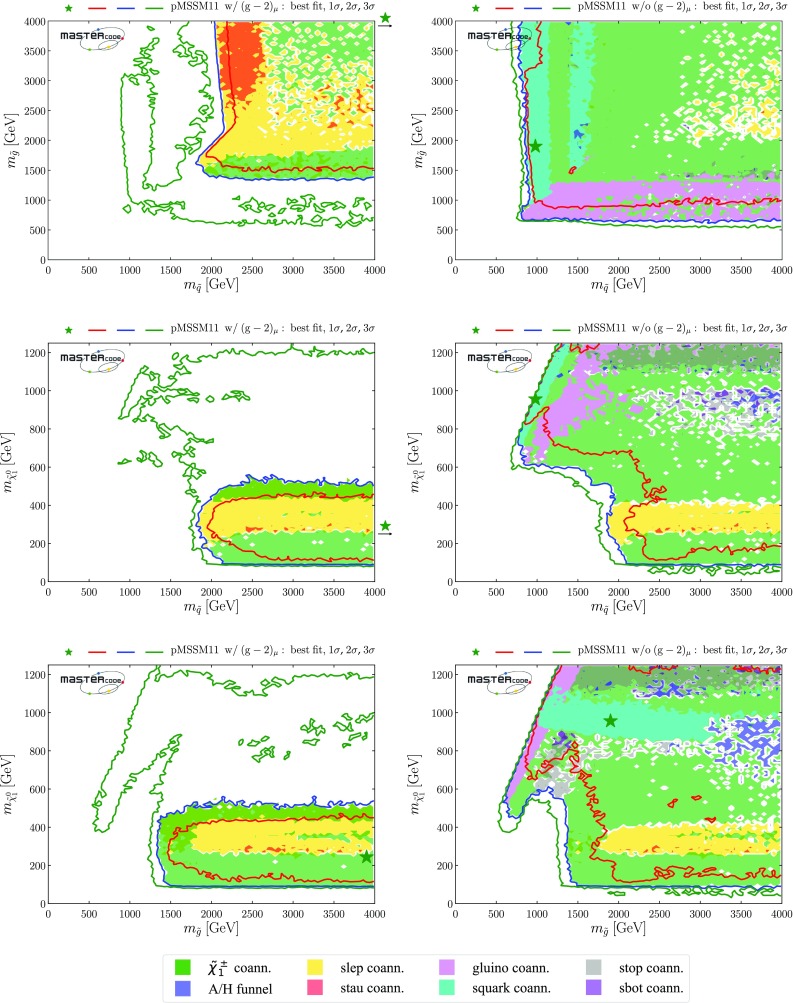
Two-dimensional projections of the global likelihood function for the pMSSM11 in the $(m_{\tilde{q}}, m_{\tilde{g}})$ planes (top panels), the $(m_{\tilde{q}}, m_{\tilde{\chi }^0_{1}})$ planes (middle panels) and the $(m_{\tilde{t}_{1}}, m_{\tilde{\chi }^0_{1}})$ planes (bottom panels), including the $(g-2)_\mu$ constraint (left panels) and dropping it (right panels)

The top row of plots in Fig. 2 show $(m_{\tilde{q}}, m_{\tilde{g}})$ planes, where $m_{\tilde{q}}$ is an average over the masses of the left- and right-handed first- and second-generation squarks, which are very similar in the pMSSM11.^22^ In the top left panel, where $(g-2)_\mu$ is included, we see 95% CL lower bounds $m_{\tilde{q}}\gtrsim 2000 \,\mathrm {GeV}$ and $m_{\tilde{g}}\gtrsim 1400 \,\mathrm {GeV}$, with regions favoured at the 68% CL appearing at slightly larger masses. We note that the best-fit point, denoted by the green star, is at large $m_{\tilde{q}}> 4000 \,\mathrm {GeV}$ and $m_{\tilde{g}}\sim 3900 \,\mathrm {GeV}$. The full set of pMSSM parameter values at this point, as well as the value of the global $\chi ^2$ function, are listed in the second column of Table 4. Important sparticle production cross-sections and decay modes at this best-fit point are shown in the top panel of Table 5.

Within the 2-$\sigma$ contour, the dominant DM mechanism is slepton coannihilation, with stau coannihilation also playing a role for $m_{\tilde{q}}\sim 2.5 \,\mathrm {TeV}$, and $\tilde{\chi }^\pm _{1}$ coannihilation playing a role at $m_{\tilde{g}}\sim 1500 \,\mathrm {GeV}$ and when $m_{\tilde{g}}\gtrsim 2500 \,\mathrm {GeV}$ and $m_{\tilde{q}}\gtrsim 2800 \,\mathrm {GeV}$. Finally, we observe that at the 3-$\sigma$ level much smaller values of $m_{\tilde{q}}$ are allowed, and that there is also a peninsula at small $m_{\tilde{g}}$ and larger $m_{\tilde{q}}$ that appears at the same level. These regions avoid the LHC exclusion searches in virtue of the same mechanisms which allow lower masses when the $(g-2)_\mu$ constraint is not applied and which will be described more in detail below. However, they are not able to satisfy the $(g-2)_{\mu }$ and this is why they take a $\Delta \chi ^2 \simeq 11$ penalty which makes them allowed only at 3-$\sigma$.

**Fig. 3:**
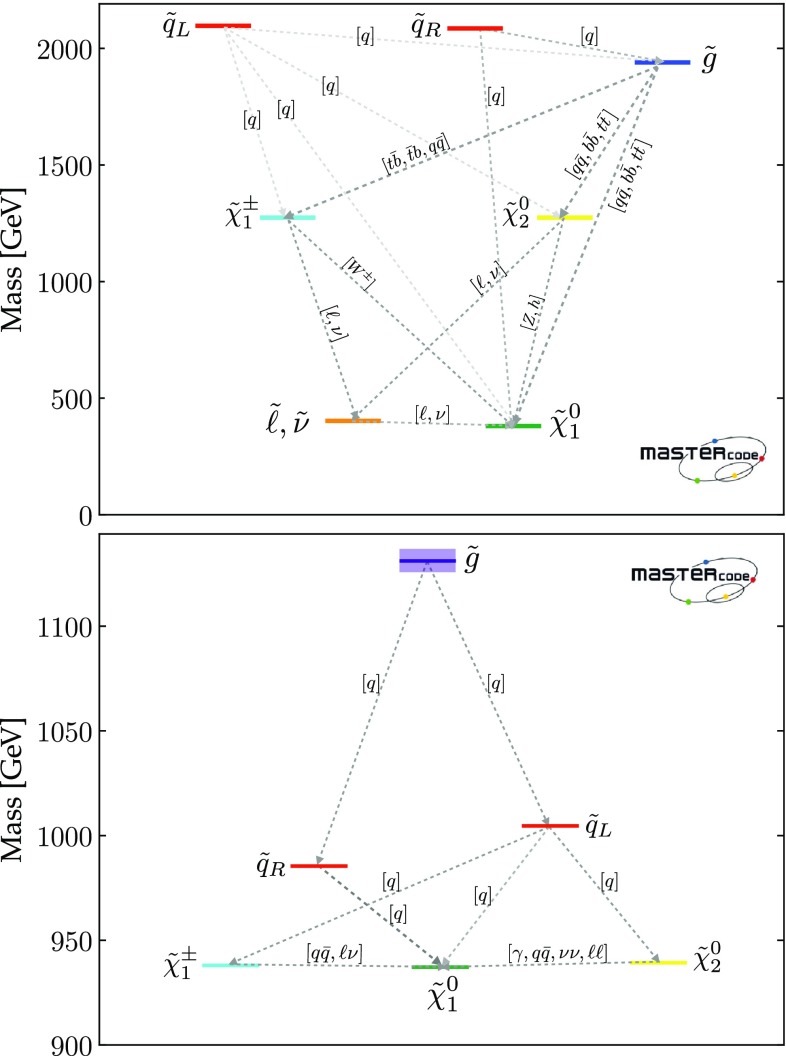
Upper panel: The dominant sparticle decay chains at the representative point in the ‘nose’ region in the top left panel of Fig. 2 (with $(g-2)_\mu$) whose parameters are listed in the second column of Table 4. Lower panel: The dominant sparticle decay chains at the representative point in the ‘nose’ region in the top right panel of Fig. 2 (without $(g-2)_\mu$) whose parameters are listed in the fourth column of Table 4 – note that the vertical scale has a suppressed zero. In both plots the widths of the sparticles are represented as semi-transparent bands around the bar representing the nominal mass value and of the same color

We also note a ‘nose’ feature corresponding to a reduction in the lower bounds when $m_{\tilde{q}}\sim 2.2 \,\mathrm {TeV}$ and $0 < m_{\tilde{q}}- m_{\tilde{g}}\lesssim 200 \,\mathrm {GeV}$. We have verified that this is due to a loss of search sensitivity when $\tilde{q}_R\rightarrow {\tilde{g}}+ q$, the *q* jet is soft, and ${\tilde{g}}\rightarrow q {\bar{q}} + {\tilde{\chi }}^*$, where ${\tilde{\chi }}^*$ denotes any electroweak ino other than the LSP, compared to a high sensitivity for $\tilde{q}_R\rightarrow q \tilde{\chi }^0_1$ in the $m_{\tilde{g}}> m_{\tilde{q}}$ case. The input pMSSM11 parameter values at a representative point in this ‘nose’ region are listed in the third column of Table 4. The upper panel of Fig. 3 displays relevant sparticle masses and the most important sparticle decay chains at this point, and numerical values are given in the second panel of Table 5. We see that the right-handed squarks decay into a variety of final states involving heavier neutralinos and charginos via intermediate gluinos due to $m_{\tilde{g}}< m_{\tilde{q}}$, reducing the effectiveness of $/ E_T$-based searches in this ‘nose’ region, compared to simple $\tilde{q}\rightarrow q + \tilde{\chi }^0_{1}$ decays.

We see significant differences in the top right panel where $(g-2)_\mu$ is dropped. The best-fit in this case is close to the 68% CL boundary at $(m_{\tilde{q}}, m_{\tilde{g}}) \sim (1000, 1600) \,\mathrm {GeV}$, with the parameters and $\chi ^2$ value shown in the fourth column of Table 4. As we discuss later, $\mathrm{BR}(B_{s, d} \rightarrow \mu ^+\mu ^-)$ and the DM density constraint play important roles in preferring a relatively low value of $m_{\tilde{q}}$. The dominant particle production and decay modes for this best-fit point are shown in the third panel of Table 5. It is notable that the 95% CL lower limits on $m_{\tilde{q}}$ and $m_{\tilde{g}}$ are reduced to $\sim 1000 \,\mathrm {GeV}$, and a less-pronounced ‘nose’ feature now appears when $m_{\tilde{q}}\sim 1 \,\mathrm {TeV}$ and $0 < m_{\tilde{g}}- m_{\tilde{q}}\lesssim 200 \,\mathrm {GeV}$. Again, we have verified that this reflects a loss of search sensitivity when ${\tilde{g}}\rightarrow \tilde{q}+ {\bar{q}}$, the ${\bar{q}}$ jet is soft, and $\tilde{q}\rightarrow q + \tilde{\chi }_{}^* (\tilde{\chi }^0_1)$, where ${\tilde{\chi }}^0_1$ is much heavier than in the fit with $(g-2)_\mu$ (for which a large SUSY contribution requires $m_{\tilde{\chi }^0_{1}}$ to be small), since the direct decay $\tilde{g} \rightarrow q \bar{q} \tilde{\chi }_{}^* (\tilde{\chi }^0_1)$ in the $m_{\tilde{q}}> m_{\tilde{g}}$ case is more sensitive than the above cascade decay in the compressed spectrum. The lower panel of Fig. 3 shows the most important sparticle decay chains at the representative point in this region whose parameters are listed in the fourth column of Table 4, and the numerical values of branching ratios are given in the bottom panel of Table 5.

The differences between the fits with and without the $(g-2)_\mu$ constraint are driven primarily by the fact that the fit with $(g-2)_\mu$ prefers small $m_{\tilde{\chi }^0_{1}}$, in which case the LHC 13-TeV searches require large $m_{\tilde{q}}$ and $m_{\tilde{g}}$, whereas the fit without $(g-2)_\mu$ favours a region with larger $m_{\tilde{\chi }^0_{1}}$. In this case, the loss of search efficiency due to a compressed spectrum allows $m_{\tilde{q}}$ and $m_{\tilde{g}}$ to be smaller than in the fit with $(g-2)_\mu$. As we see later, in this compressed region the LSP is mainly a neutral Higgsino, and coannihilations with a nearby charged Higgsino and the $\tilde{\chi }^0_{2}$ are important in determining the relic neutralino density. Coannihilations with first- and second-generation squarks are also relevant here and in a band with $m_{\tilde{q}}\sim 1 \,\mathrm {TeV}\lesssim m_{\tilde{g}}$ (coloured cyan), whereas coannihilations with gluinos are important along a band with $(1 \,\mathrm {TeV}, 2 \,\mathrm {TeV}) \ni m_{\tilde{g}}\lesssim m_{\tilde{q}}$ (coloured magenta). In this plane the 1-, 2- and 3-$\sigma$ contours lie relatively close to each other.

In the middle row of Fig. 2 we display the corresponding $(m_{\tilde{q}}, m_{\tilde{\chi }^0_{1}})$ planes. We see a preference for $m_{\tilde{\chi }^0_{1}} \lesssim 550 \,\mathrm {GeV}$ in the left panel, where the $(g-2)_\mu$ constraint is included, whereas much larger values of $m_{\tilde{\chi }^0_{1}}$ are allowed at the 3-$\sigma$ level. These larger values of $m_{\tilde{\chi }^0_{1}}$ appear within the 1- and 2-$\sigma$ contours in the middle right panel where the $(g-2)_\mu$ constraint is dropped. We also see again that larger values of $m_{\tilde{q}}$ are favoured when $(g-2)_\mu$ is included, whereas a small $m_{\tilde{q}}- m_{\tilde{\chi }^0_{1}}$ mass difference is preferred when the $(g-2)_\mu$ constraint is dropped. In both the middle panels the dominant DM mechanisms are slepton and $\tilde{\chi }^\pm _{1}$ coannihilation, with the rapid annihilation via the heavy *H* / *A* Higgs bosons becoming important at large masses when $(g-2)_\mu$ is dropped. Similar features are seen in the $(m_{\tilde{g}}, m_{\tilde{\chi }^0_{1}})$ planes displayed in the bottom row of Fig. 2.

**Table 5:** Dominant particle production and decay modes for various pMSSM11 parameter sets. Top panel: best-fit point with $(g-2)_\mu$. Second panel: representative point in the ‘nose’ region in fit with $(g-2)_\mu$. Third panel: best-fit point without $(g-2)_\mu$. Bottom panel: representative point in the ‘nose’ region in fit without $(g-2)_\mu$

*Dominant sparticle production and decay modes at best-fit point with* $(g-2)_\mu$	
Production	$\sigma$ [fb]
$pp \rightarrow \tilde{t}_1 \tilde{t}_1 + \hbox {X}$	0.25
$pp \rightarrow \tilde{b}_1 \tilde{b}_1 + \hbox {X}$	0.13

Decays (mass [GeV])	BR [%]
$\tilde{t}_1 (1481) \rightarrow b \tilde{\chi }_1^\pm (270) /t \tilde{\chi }_2^0 (270) /t \tilde{\chi }_1^0 (249)$	56 / 25 / 19
$\tilde{b}_1 (1586) \rightarrow t \tilde{\chi }_1^\pm (270) /b \tilde{\chi }_2^0 (270) /b \tilde{\chi }_{3/4}^0 (270) /b \tilde{\chi }_1^0 (249)$	60 / 29 / 5 / 4
$\tilde{\chi }_1^\pm (270) \rightarrow \ell ^\pm \nu _\ell \tilde{\chi }_1^0 (249) /q q^\prime \tilde{\chi }_1^0(249) /\tau ^\pm \nu _\tau \tilde{\chi }_1^0 (249)$	52 / 38 / 1
$\tilde{\chi }_2^0 (270) \rightarrow \nu \bar{\nu }\tilde{\chi }_1^0 (249) /\ell ^\pm \ell ^\mp \tilde{\chi }_1^0 (249) /\tau ^\pm \tau ^\mp \tilde{\chi }_1^0 (249)$	53 / 37 / 1

*Dominant sparticle production and decay modes at ‘nose’ point in fit with* $(g-2)_\mu$	
Production	$\sigma$ [fb]
$pp \rightarrow \tilde{q} \tilde{q} + \hbox {X}$	3.4
$pp \rightarrow \tilde{g} \tilde{q} + \hbox {X}$	3.4
$pp \rightarrow \tilde{g} \tilde{g} + \hbox {X}$	0.5

Decays (mass [GeV])	BR [%]
$\tilde{g} (1942) \rightarrow q q \tilde{\chi }_1^0 (380)/q q^\prime \tilde{\chi }_1^\pm (1273) \,/\, q q \tilde{\chi }_2^0 (1273)$	45 / 37 / 18
$\tilde{q}_L (2099) \,\rightarrow \, q \tilde{\chi }_1^\pm (1273)/q \tilde{g} (1942)/q \tilde{\chi }_2^0 (1273)/q \tilde{\chi }_1^0 (380)$	48 / 26 / 24 / 2
$\tilde{q}_R (2086) \rightarrow q \tilde{g} (1942)/q \tilde{\chi }_1^0 (380)$	57 / 43
$\tilde{\chi }_1^\pm (1273) \rightarrow [\ell ^\pm \tilde{\nu }_\ell (400) \rightarrow \ell ^\pm \nu _\ell \tilde{\chi }_1^0(380)] / [\nu _\ell \tilde{\ell }^\pm (404) \rightarrow \nu _\ell \ell ^\pm \tilde{\chi }_1^0(380)]$	50 / 50
$\tilde{\chi }_2^0 (1273) \rightarrow [\ell ^\pm \tilde{\ell }^\mp (404) \rightarrow \ell ^+ \ell ^- \tilde{\chi }_1^0(380)] / [\nu \tilde{\nu }_\ell (400) \rightarrow \nu _\ell \nu _\ell \tilde{\chi }_1^0(380)]$	50 / 50

*Dominant sparticle production and decay modes at best-fit point without* $(g-2)_\mu$	
Production	$\sigma$ [fb]
$pp \rightarrow \tilde{q} \tilde{q} + \hbox {X}$	386
$pp \rightarrow \tilde{g} \tilde{q} + \hbox {X}$	51
$pp \rightarrow \tilde{g} \tilde{g} + \hbox {X}$	1

Decays (mass [GeV])	BR [%]
$\tilde{g} (1908) \rightarrow q \tilde{q}_R (988) / q \tilde{q}_L (1008)$	51 / 49
$\tilde{q}_L (1008) \rightarrow q \tilde{\chi }_1^\pm (955) / q \tilde{\chi }_1^0 (954) / q \tilde{\chi }_2^0 (954)$	55 / 39 / 6
$\tilde{q}_R (988) \rightarrow q \tilde{\chi }_2^0 (954) / q \tilde{\chi }_1^0 (954)$	98 / 2

*Dominant sparticle production and decay modes at ‘nose’ point in fit without* $(g-2)_\mu$	
Production	$\sigma$ [fb]
$pp \rightarrow \tilde{q} \tilde{q} + \hbox {X}$	619
$pp \rightarrow \tilde{g} \tilde{q} + \hbox {X}$	586
$pp \rightarrow \tilde{g} \tilde{g} + \hbox {X}$	87

Decays (mass [GeV])	BR [%]
$\tilde{g} (1131) \rightarrow q \tilde{q}_R (984) / q \tilde{q}_L (1003)$	44 / 56
$\tilde{q}_L (1003) \rightarrow q \tilde{\chi }_1^\pm (939) / q \tilde{\chi }_1^0 (937) / q \tilde{\chi }_2^0 (938)$	58 / 38 / 4
$\tilde{q}_R (984) \rightarrow q \tilde{\chi }_2^0 (938) / q \tilde{\chi }_1^0 (937)$	96 / 4

*Third-generation squarks*

**Fig. 4:**
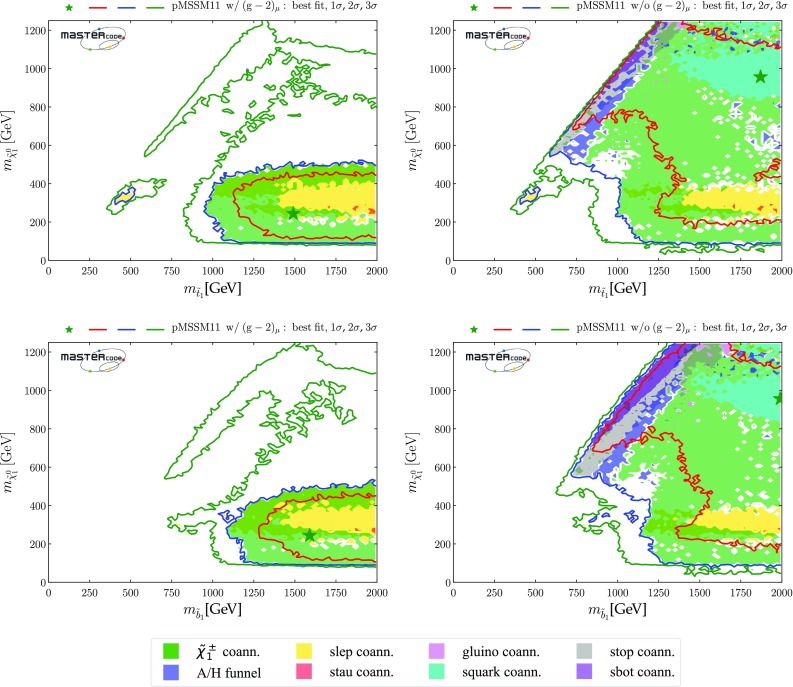
Two-dimensional projections of the global likelihood function for the pMSSM11 in the $(m_{\tilde{t}_{1}}, m_{\tilde{\chi }^0_{1}})$ planes (upper panels) and the $(m_{\tilde{b}_1}, m_{\tilde{\chi }^0_{1}})$ planes (lower panels), including the $(g-2)_\mu$ constraint (left panels) and dropping it (right panels)

Figure 4 displays the $(m_{\tilde{t}_{1}}, m_{\tilde{\chi }^0_{1}})$ planes in the upper panels and the $(m_{\tilde{b}_1}, m_{\tilde{\chi }^0_{1}})$ planes in the lower panels, again including the $(g-2)_\mu$ constraint in the left panels and dropping it in the right panels. We see that both the third-generation squark masses may be considerably smaller than those in the first two generations. Specifically, an isolated, low stop-mass region where $(m_{\tilde{t}_{1}}, m_{\tilde{\chi }^0_{1}}) \sim (500, 300) \,\mathrm {GeV}$ is allowed at the 95% CL^23^ in both the cases with and without $(g-2)_\mu$, which is connected in the latter case to the rest of the 95% CL region at the 3-$\sigma$ level. The low stop-mass island is allowed and defined by different physics mechanisms. First, the third-generation-squark spectra are sufficiently compressed to allow the points to bypass the LHC13 constraints. Moreover, it is characterized by compressed-slepton spectra as well, which explains the fact that the region is shaded in yellow in the plots. We also note that it can not be extended to lower stop masses because otherwise it would be disallowed by sbottom searches, since in our scenario the masses of the stop and sbottom squarks are defined by a single soft SUSY-breaking mass term and the sbottoms would not be sufficiently compressed to be allowed by LHC searches. LHC constraints also limit its extensions in the direction of lower neutralino (too light third-generation squarks) or higher stop masses (due to the loss of compression). Finally, at heavier neutralino masses slepton coannihilation is insufficient to reduce the relic density into the allowed range.4 When $(g-2)_\mu$ is dropped, extended 95% CL regions with $m_{\tilde{\chi }^0_{1}} \gtrsim 500 \,\mathrm {GeV}$ appear when $m_{\tilde{t}_{1}} \gtrsim 1100 \,\mathrm {GeV}$ and $m_{\tilde{b}_1} \gtrsim 1250 \,\mathrm {GeV}$. When $(g-2)_\mu$ is included, there are extended regions with $m_{\tilde{\chi }^0_{1}} \gtrsim 500 \,\mathrm {GeV}$ that appear at the 3-$\sigma$ level. Within the 1- and 2-$\sigma$ contours, the dominant DM mechanisms are slepton and $\tilde{\chi }^\pm _{1}$ coannihilation, with rapid annihilation via the heavy *H* / *A* Higgs bosons again becoming important at large $m_{\tilde{\chi }^0_{1}}$ when $(g-2)_\mu$ is dropped. The same mechanism is also active inside the white regions between $800 \,\mathrm {GeV}~(1 \,\mathrm {TeV}) \lesssim m_{\tilde{t}_{1}}~(m_{\tilde{b}_{1}}) \lesssim 1.1~(1.2) \,\mathrm {TeV}$ and $400 \,\mathrm {GeV}\lesssim m_{\tilde{\chi }^0_{1}} \lesssim 600 \,\mathrm {GeV}$, the blue shading being absent due to the proxy-measure being not sufficiently descriptive in this parameter space region. Stop and sbottom coannihilation are also important for small $m_{\tilde{t}_{1}} - m_{\tilde{\chi }^0_{1}}$ and $m_{\tilde{b}_{1}} - m_{\tilde{\chi }^0_{1}}$.

*Sleptons*

**Fig. 5:**
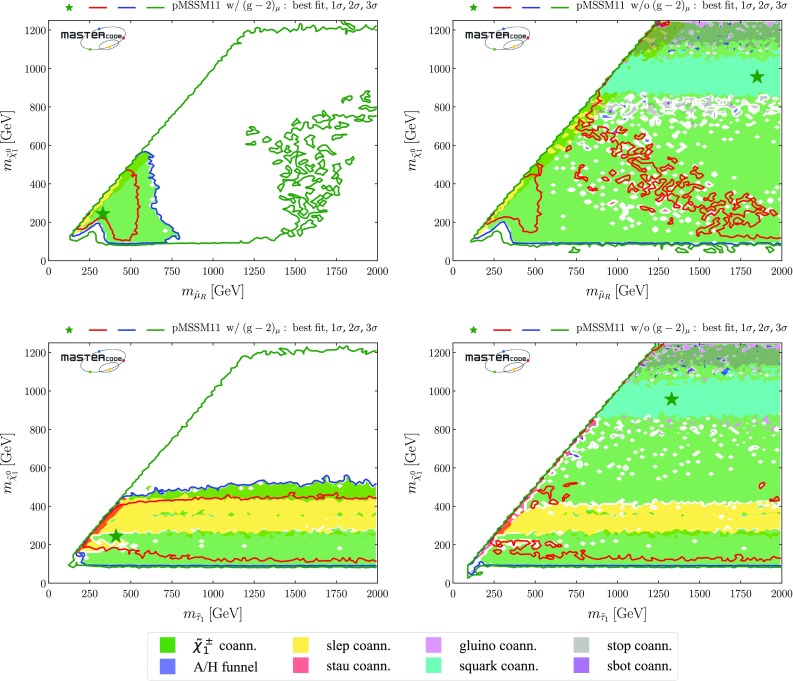
Two-dimensional projections of the global likelihood function for the pMSSM11 in the $(m_{\mu _R}, m_{\tilde{\chi }^0_{1}})$ planes (upper panels) and the $(m_{\tilde{\tau }_1}, m_{\tilde{\chi }^0_{1}})$ planes (lower panels), including the $(g-2)_\mu$ constraint (left panels) and dropping it (right panels)

As was to be expected, there are large differences between the $(m_{\tilde{\mu }_R}, m_{\tilde{\chi }^0_{1}})$ planes with and without the $(g-2)_\mu$ constraint, shown in the upper panels in Fig. 5. We see in the upper left plane a preference for $m_{\tilde{\mu }_R} \lesssim 550 (750) \,\mathrm {GeV}$ and $m_{\tilde{\chi }^0_{1}} \lesssim 500 (550) \,\mathrm {GeV}$ at the 68 (95)% CL, enforced by the $(g-2)_\mu$ constraint, with larger masses allowed at the 3-$\sigma$ level. There is also a 68% CL region with similar ranges of $m_{\tilde{\mu }_R}$ and $m_{\tilde{\chi }^0_{1}}$ in the case without $(g-2)_\mu$ (upper right panel), but the 95% CL region extends to much larger values of $m_{\tilde{\mu }_R}$ and $m_{\tilde{\chi }^0_{1}}$, and there is also a second, extended 68% CL region that is separated by a band of points with only slightly higher $\chi ^2$. In both these plots, we see a very narrow strip where slepton-$\tilde{\chi }^0_{1}$ coannihilation is important, whereas $\tilde{\chi }^\pm _{1}$ coannihilation dominates in most of the regions allowed at the 95% CL, supplemented by annihilation via the *H* / *A* bosons at large $m_{\tilde{\chi }^0_{1}}$ when $(g-2)_\mu$ is dropped. We do not display the corresponding $(m_{\tilde{\mu }_L}, m_{\tilde{\chi }^0_{1}})$ and $(m_{\tilde{e}_{L,R}}, m_{\tilde{\chi }^0_{1}})$ planes, which are very similar because we impose universality on the soft SUSY-breaking masses of the first two slepton generations.

However, in the pMSSM11 the soft SUSY-breaking stau masses are allowed to be different, with the result seen in the lower panels of Fig. 5 that large values of $m_{\tilde{\tau }_1}$ are allowed at the 68 and 95% CL even when $(g-2)_\mu$ is imposed. The main differences between the cases with and without $(g-2)_\mu$ are that larger values of $m_{\tilde{\chi }^0_{1}}$ are allowed in the latter case - indeed, the best-fit point has $m_{\tilde{\tau }_1}\sim m_{\tilde{\chi }^0_{1}} \sim 1 \,\mathrm {TeV}$. We see, once again, the importance of the slepton and $\tilde{\chi }^\pm _{1}$ coannihilation mechanisms, supplemented by annihilation via *H* / *A* at large $m_{\tilde{\chi }^0_{1}}$ in the case without $(g-2)_\mu$. The small ‘nose’ at $(m_{\tilde{\tau }_1}, m_{\tilde{\chi }^0_{1}}) \sim (100, 50) \,\mathrm {GeV}$ is a remnant of rapid annihilations via direct-channel *Z* and *h*(125) poles.

*Electroweak inos*

**Fig. 6:**
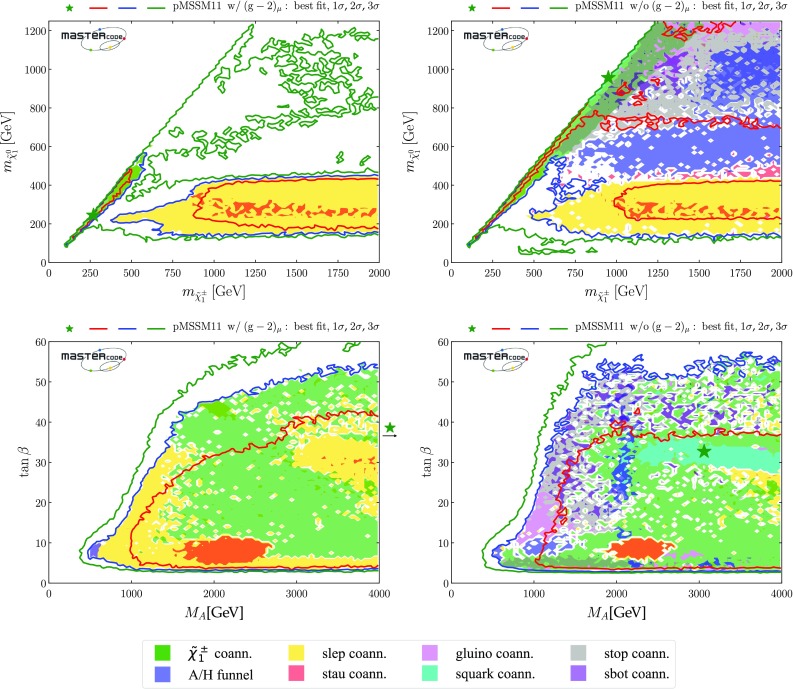
Two-dimensional projections of the global likelihood function for the pMSSM11 in the $(m_{\tilde{\chi }^\pm _{1}}, m_{\tilde{\chi }^0_{1}})$ planes (upper panels) and the $(M_A, \tan \beta )$ planes (lower panels), including the $(g-2)_\mu$ constraint (left panels) and dropping it (right panels)

In the upper panels of Fig. 6 we show the $(m_{\tilde{\chi }^\pm _{1}}, m_{\tilde{\chi }^0_{1}})$ planes with (left panel) and without (right panel) the $(g-2)_\mu$ constraint. In both panels we see a $\tilde{\chi }^\pm _{1}$ coannihilation strip starting at $(m_{\tilde{\chi }^\pm _{1}}, m_{\tilde{\chi }^0_{1}}) \sim (100, 100) \,\mathrm {GeV}$, and extending to larger $m_{\tilde{\chi }^\pm _{1}}$ in the latter case. This $\tilde{\chi }^\pm _{1}$ coannihilation strip is isolated in the $(g-2)_\mu$ case, but connected to an extended 95% CL region at large $m_{\tilde{\chi }^\pm _{1}}$ in the case without $(g-2)_\mu$. In both panels there is a broad band with $m_{\tilde{\chi }^0_{1}} \sim 150$ to $400 \,\mathrm {GeV}$ where slepton coannihilation dominates. A major difference between the plots is the extensive region at large $m_{\tilde{\chi }^0_{1}}$ in the case without $(g-2)_\mu$ where annihilation via *H* / *A* is important. The best-fit points are at $m_{\tilde{\chi }^\pm _{1}} \sim m_{\tilde{\chi }^0_{1}} \sim 250 \,\mathrm {GeV}$ in the $(g-2)_\mu$ case and $\sim 1000 \,\mathrm {GeV}$ in the case without it.

*Heavy Higgs bosons*

The 68 and 95% CL regions in the pair of $(M_A, \tan \beta )$ planes shown in the lower panels of Fig. 6 display the importance of the latest ATLAS constraint on $A/H \rightarrow \tau ^+ \tau ^-$ decays with $\sim 36$/fb of data at 13 TeV [127], which disfavours regions with $M_A\lesssim 1 \,\mathrm {TeV}$ at larger $\tan \beta$. We also note that the dominant DM mechanisms display significant differences. Chargino coannihilation is important in both planes, but slepton coannihilation appears only in the case where $(g-2)_\mu$ is included. In this case annihilation via the *H* / *A* poles appears only when $M_A\lesssim 1 \,\mathrm {TeV}$, but it appears also at larger $M_A$ when $(g-2)_\mu$ is dropped. We see in both cases a limited region with $M_A\sim 2 \,\mathrm {TeV}$ and $\tan \beta \lesssim 10$ where stau coannihilation dominates. In our previous pMSSM10 analysis [13] the interplay of the LHC electroweak searches, $(g-2)_\mu$ and the DM constraints, heavily relying on the fact that only one independent slepton mass parameter was allowed, led to a region with $25 \lesssim \tan \beta \lesssim 45$ being preferred at the 68% CL. However, in the pMSSM11, dropping the restriction $m_{\tilde{\tau }_{\ }}= {m_{\tilde{\ell }}}$ now allows values of $\tan \beta < 5$ for a wide range of $M_A$ values. Also, despite the updated (stronger) constraints on $H/A \rightarrow \tau \tau$, values down to $M_A\sim 500 \,\mathrm {GeV}$ are still allowed at the 95% CL.

## One-dimensional likelihood functions

In this section we present the profile $\chi ^2$ likelihood functions corresponding to various one-dimensional projections of the results from our global fits, again comparing those with and without the $(g-2)_\mu$ constraint. In the following series of plots, results including the LHC 13-TeV constraints are shown as solid lines, and those using only 8-TeV results are shown as dashed lines. Results obtained including $(g-2)_\mu$ are shown in blue and those obtained without $(g-2)_\mu$ are shown in green.

### $(g-2)_\mu$

**Fig. 7:**
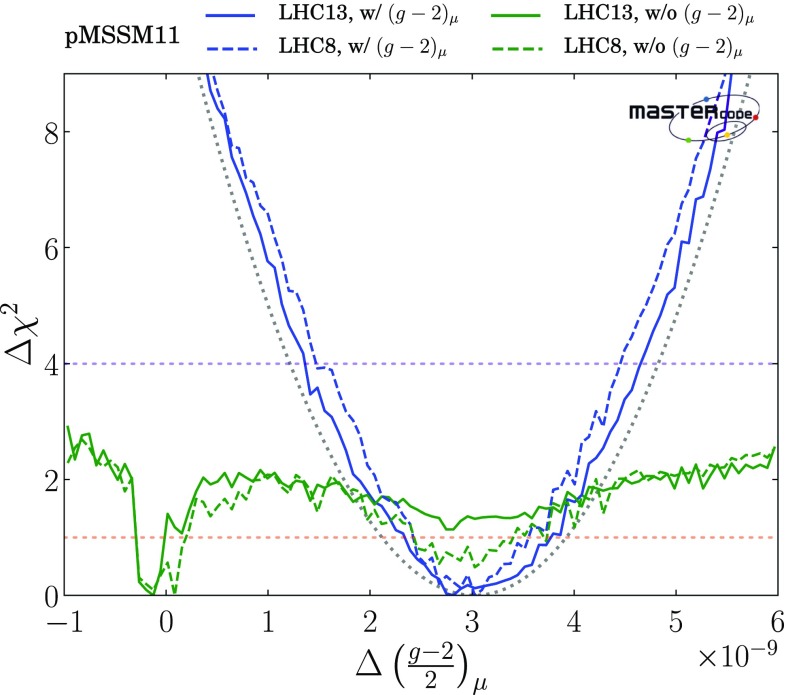
One-dimensional profile likelihood functions for $(g-2)_\mu$ in the pMSSM11, with (blue) and without (green) applying the $(g-2)_\mu$ constraint *a priori* and with (solid) and without (dashed) applying the constraints coming from the LHC run at 13 TeV. Also shown as a dotted line is the experimental constraint [108, 109], taking into account the theoretical uncertainty [100–107] within the Standard Model

As a preliminary, Fig. 7 shows the one-dimensional profile likelihood functions for $(g-2)_\mu$ with (blue) and (green) without applying the $(g-2)_\mu$ constraint *a priori*. Comparing the solid and dashed lines, we see very little difference between the results using and discarding the LHC 13-TeV data. The results including $(g-2)_\mu$ (blue lines) largely reflect our implementation of the $(g-2)_\mu$ constraint shown in Table 2. Interestingly, when this constraint is not applied *a priori* (green lines), whilst a very small SUSY contribution to $(g-2)_\mu$ is preferred, a wide range of values of $(g-2)_\mu$ are found to be allowed at the $\Delta \chi ^2 {\sim 2}$ level and the experimental value can be accommodated at the 1.5-$\sigma$ level. Although the other data certainly do not favour a large SUSY contribution to $(g-2)_\mu$, neither do they exclude it.

### Sparticle masses

*Squarks and gluinos*

**Fig. 8:**
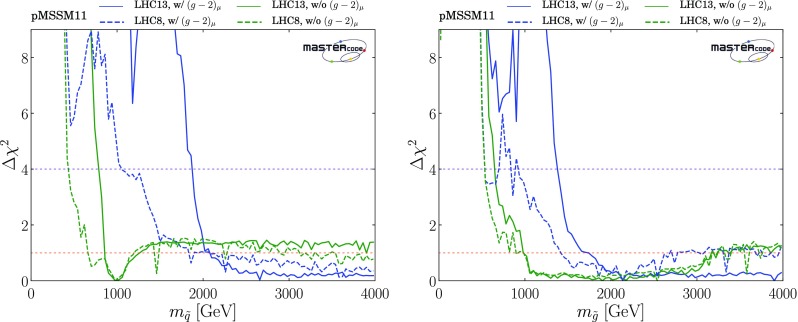
Left panel: One-dimensional profile likelihood functions for the $\tilde{q}$ mass in the pMSSM11 with (blue) and without the $(g-2)_\mu$ constraint (green) and with (solid) and without (dashed) applying the constraints from LHC Run II. Right panel: Similarly for the ${\tilde{g}}$ mass

The profile likelihood functions for squarks and gluinos are shown in Fig. 8. The left panel is for $m_{\tilde{q}}$, where we see that when the 13-TeV LHC data and $(g-2)_\mu$ constraint are included (solid blue line), there is a monotonic decrease in $\chi ^2$ as ${m_{\tilde{q}}}$ increases, with $m_{\tilde{q}}\gtrsim 1.9 \,\mathrm {TeV}$ at the 95% CL (horizontal dotted line). This constraint is much stronger than that obtained with 8-TeV data alone (dashed blue and green lines): $m_{\tilde{q}}\gtrsim 1.0 \,\mathrm {TeV}$ at the 95% CL. In particular, the 13-TeV data exclude a squark coannihilation strip that had been allowed by the 8-TeV data. When $(g-2)_\mu$ is dropped but the 13-TeV data retained (solid green line), the $\chi ^2$ function exhibits a global minimum at $m_{\tilde{q}}\sim 1 \,\mathrm {TeV}$, with a plateau at $\Delta \chi ^2 \simeq 1.5$ at larger $m_{\tilde{q}}$. Important roles in the location of this global minimum are played by the $\mathrm{BR}(B_{s, d} \rightarrow \mu ^+\mu ^-)$ constraint as discussed in Sect. 4.4, whose contribution to the global $\chi ^2$ function at this point is $\sim 1.1$ lower than at large $m_{\tilde{q}}$, and by the relic DM density constraint, which is satisfied thanks to multiple coannihilation processes as discussed in Sect. 4.6.

In the right panel of Fig. 8 for $m_{\tilde{g}}$, we see that with both the LHC 13-TeV data and $(g-2)_\mu$ included $m_{\tilde{g}}\gtrsim 1.8 \,\mathrm {TeV}$ (solid blue line), whereas without $(g-2)_\mu$ we find $m_{\tilde{g}}\gtrsim 1.0 \,\mathrm {TeV}$ (solid green line). On the other hand, in the absence of the LHC 13-TeV data (dashed lines), $m_{\tilde{g}}\gtrsim 500 \,\mathrm {GeV}$ would have been allowed at the 95% CL, whether $(g-2)_\mu$ is included, or not. The LHC 13-TeV run has excluded a region of gluino coannihilation that was allowed by the 8-TeV data.

*Third-generation squarks*

**Fig. 9:**
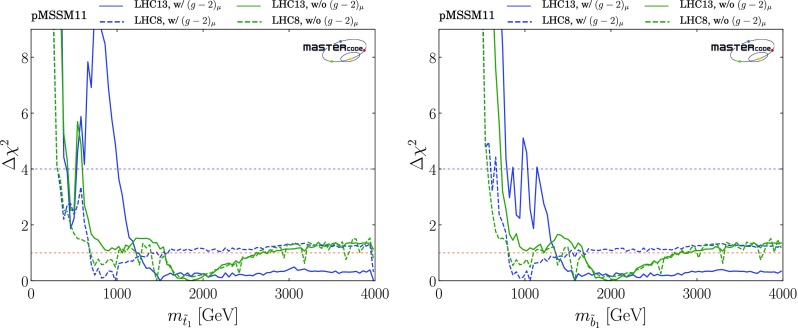
Left panel: One-dimensional profile likelihood functions for the $\tilde{t}_1$ mass in the pMSSM11 with (blue) and without the $(g-2)_\mu$ constraint (green) and with (solid) and without (dashed) applying the constraints from LHC Run II. Right panel: Similarly for the $\tilde{b}_1$ mass

An analogous pair of plots showing the profile likelihood functions for the masses of the $\tilde{t}_{1}$ and $\tilde{b}_{1}$ are shown in the left and right panels of Fig. 9. When the LHC 13-TeV data are included we see in the left panel a well-defined local minimum of the $\chi ^2$ function in a compressed-stop region with $\Delta \chi ^2 \sim 2.3$ for $m_{\tilde{t}_{1}} \sim 400 \,\mathrm {GeV}$. This is followed by a local maximum that exceeds $\Delta \chi ^2 > 9$ for $m_{\tilde{t}_{1}} \sim 800 \,\mathrm {GeV}$ when $(g-2)_\mu$ is included (solid blue line) but is lower when $(g-2)_\mu$ is dropped (solid green line). This is followed in both cases by a monotonic decrease for larger $m_{\tilde{t}_{1}}$ and a global minimum of $\chi ^2$ for $m_{\tilde{t}_{1}} \sim 1800 \,\mathrm {GeV}$.

In the case of $m_{\tilde{b}_{1}}$ (right panel of Fig. 9). when the 13-TeV LHC data and $(g-2)_\mu$ are included (solid blue line) there are some irregularities in the $\chi ^2$ function for $m_{\tilde{b}_{1}} \sim 1000 \,\mathrm {GeV}$, but no hint of a compressed-sbottom region when $(g-2)_\mu$ is dropped (dashed blue line). Comparing with the situation when only LHC 8-TeV used, we see that the 13-TeV data have increased significantly the pressure on scenarios with $m_{\tilde{b}_{1}} \lesssim 1.5 \,\mathrm {TeV}$. At larger masses the $\chi ^2$ functions $m_{\tilde{b}_{1}}$ are very similar to those for $m_{\tilde{t}_{1}}$, whether $(g-2)_\mu$ is included or not.

*Sleptons*

**Fig. 10:**
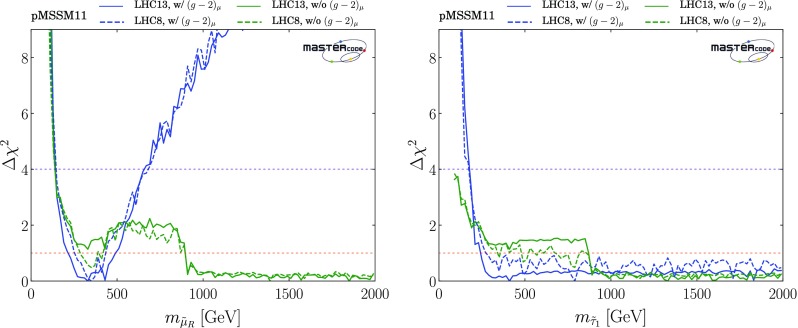
Left panel: One-dimensional profile likelihood functions for the $\tilde{\mu }_R$ mass in the pMSSM11 with (blue) and without the $(g-2)_\mu$ constraint (green) and with (solid) and without (dashed) applying the constraints from LHC Run II. Right panel: Similarly for the $\tilde{\tau }_1$ mass

Figure 10 displays analogous plots of the profile likelihood functions for $m_{\tilde{\mu }_R}$ (left panel, those for $m_{\tilde{\mu }_L}$ and $m_{\tilde{e}_{L,R}}$ are very similar) and $m_{\tilde{\tau }_1}$ (right panel, that for $m_{\tilde{\tau }_2}$ is quite similar). When the $(g-2)_\mu$ constraint is implemented (blue lines), the $\chi ^2$ function for $m_{\tilde{\mu }_R}$ exhibits the expected well-defined minimum at $m_{\tilde{\mu }_R} \sim 200$ to $500 \,\mathrm {GeV}$ when the LHC 13-TeV data are included. In the absence of the $(g-2)_\mu$ constraint (green lines), this is replaced by a plateau with $\Delta \chi ^2 \sim 2$ that extends to $m_{\tilde{\mu }_R} \sim 900 \,\mathrm {GeV}$, where the profile likelihood function drops to very small values for larger $m_{\tilde{\mu }_R}$. The drop occurs because this fit prefers $m_{\tilde{\chi }^0_{1}} \sim 900$ to $1000 \,\mathrm {GeV}$, and any heavier ${\tilde{\mu }_R}$ can decay into a $\tilde{\chi }^0_{1}$ in this mass range.

**Fig. 11:**
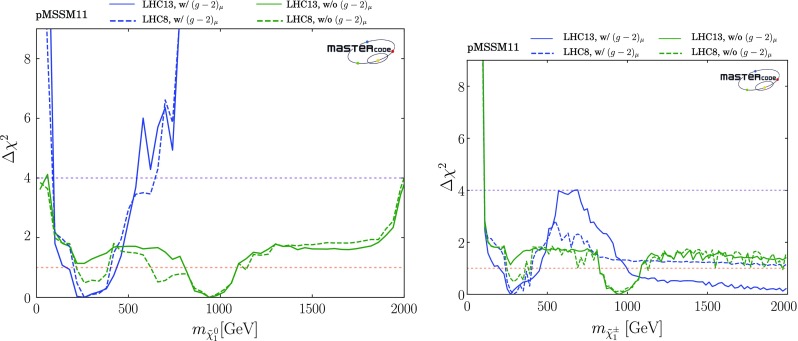
Left panel: One-dimensional profile likelihood functions for the $\tilde{\chi }^0_{1}$ mass in the pMSSM11 with (blue) and without the $(g-2)_\mu$ constraint (green) and with (solid) and without (dashed) applying the constraints from LHC Run II. Right panel: Similarly for the $\tilde{\chi }^\pm _{1}$ mass

We see in the right panel of Fig. 10 that when $(g-2)_\mu$ is included (blue lines) the profile likelihood function for $m_{\tilde{\tau }_1}$ is quite different from that for $m_{\tilde{\mu }_R}$, thanks to the decoupling between their soft SUSY-breaking masses in the pMSSM11. The $\chi ^2$ function falls monotonically to a local minimum when $m_{\tilde{\tau }_1}\sim 300 \,\mathrm {GeV}$ and remains small for larger $m_{\tilde{\tau }_1}$, whether the LHC 13-TeV data are included (solid line), or not (dashed line). However, when $(g-2)_\mu$ is dropped (green lines), the profile likelihood function for $m_{\tilde{\tau }_1}$ is quite similar to that for $m_{\tilde{\mu }_R}$, also exhibiting a plateau with $\Delta \chi ^2 \sim 2$ and falling to small values for $m_{\tilde{\tau }_1}\gtrsim 900 \,\mathrm {GeV}$ when the LHC 13-TeV data are included. This feature appears because, in order to avoid a charged LSP, a smaller value of $m_{\tilde{\tau }_1}$ would require a smaller value of $m_{\tilde{\chi }^0_{1}}$, which is disfavoured as seen in the left panel of Fig. 11 and discussed below.

*Electroweak inos*

Figure 11 shows the profile likelihood functions for the lightest neutralino $\tilde{\chi }^0_{1}$ (left panel) and the lighter chargino $\tilde{\chi }^\pm _{1}$ (right panel). When the $(g-2)_\mu$ constraint is applied (blue lines), the $\chi ^2$ function for $m_{\tilde{\chi }^0_{1}}$ including 13-TeV data exhibits a well-defined but broad minimum at $m_{\tilde{\chi }^0_{1}} \sim 100$ to $400 \,\mathrm {GeV}$. This preference for small $m_{\tilde{\chi }^0_{1}}$ was already seen in the upper boundaries of the 68% and 95% CL regions in the planes involving $m_{\tilde{\chi }^0_{1}}$ shown in the previous section when the $(g-2)_\mu$ constraint is applied (left panels).

On the other hand, when the $(g-2)_\mu$ constraint is dropped (green lines) we see a preference for $m_{\tilde{\chi }^0_{1}} \sim 950 \,\mathrm {GeV}$. Despite the fact that the LSP is a nearly-pure Higgsino at this best-fit point, this mass of $\sim 950$ GeV is below the $\sim 1.1 \,\mathrm {TeV}$ mass expected for a Higgsino dark matter candidate. This arises because, at the best-fit point, several of the squark masses lie close to the LSP mass, making multiple coannihilation important. Due to the relatively large number of states with masses close to the Higgsino, their density actually increases the final LSP relic density,^24^ thereby pushing the mass of the Higgsino below its nominal $\sim 1.1 \,\mathrm {TeV}$ value.

Turning now to the profile likelihood functions for the lighter chargino $\tilde{\chi }^\pm _{1}$ (right panel of Fig. 11), we see that when $(g-2)_\mu$ is taken into account (blue lines) the $\chi ^2$ function also features a well-defined minimum for $m_{\tilde{\chi }^\pm _{1}} \sim 200$ to $500 \,\mathrm {GeV}$ (that for $\tilde{\chi }^0_{2}$ is very similar), reflecting the importance of $\tilde{\chi }^\pm _{1} - \tilde{\chi }^0_{1}$ coannihilation. This minimum is followed by a rise to a local maximum at $m_{\tilde{\chi }^\pm _{1}} \sim 600 \,\mathrm {GeV}$, which is more pronounced when the 13-TeV data are included (solid blue), followed by a slow decrease as $m_{\tilde{\chi }^\pm _{1}}$ increases further. When the $(g-2)_\mu$ constraint is dropped and the LHC 13-TeV data are included (solid green line), the $\chi ^2$ functions for $m_{\tilde{\chi }^\pm _{1}}$ and $m_{\tilde{\chi }^0_{2}}$ have global minima at $m_{\tilde{\chi }^0_{1}} \sim 1000 \,\mathrm {GeV}$, accompanied by plateaus with $\Delta \chi ^2 \sim 2$ at smaller and larger values of $m_{\tilde{\chi }^\pm _{1}}$. The dip in the $\chi ^2$ function occurs because the fit to $\mathrm{BR}(B_{s, d} \rightarrow \mu ^+\mu ^-)$ is improved for $m_{\tilde{\chi }^\pm _{1}} \simeq m_{\tilde{\chi }^0_{2}} \sim m_{\tilde{\chi }^0_{1}} \sim 1 \,\mathrm {TeV}$. Chargino coannihilation is important around this global minimum of the $\chi ^2$ function, and so are other coannihilation mechanisms, as we discuss later.

### Neutralino composition

**Fig. 12:**
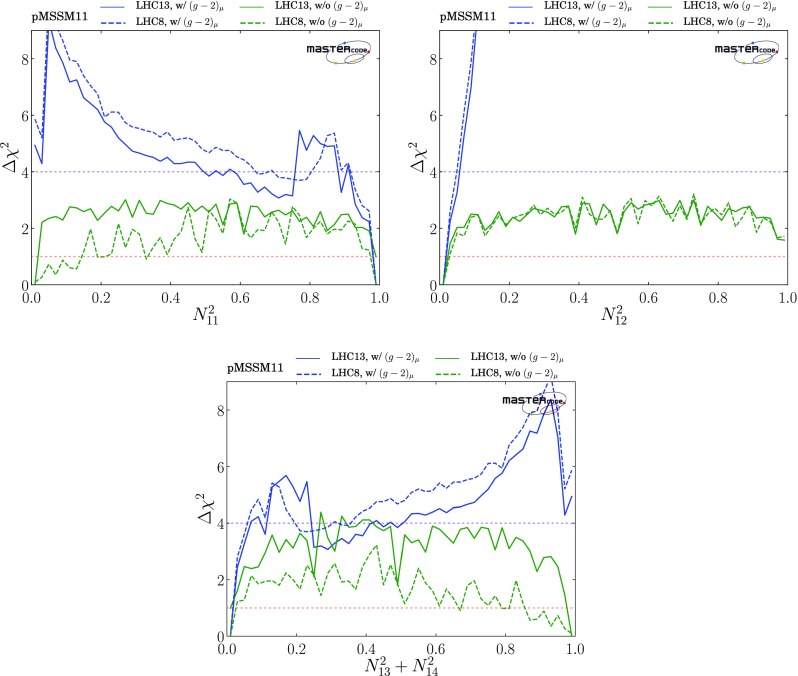
One-dimensional likelihood plots for the $\tilde{B}$ fraction in the LSP $\tilde{\chi }^0_{1}$ composition in the (upper left), for the $\tilde{W}^3$ fraction (upper right) and for the $\tilde{H}_{u,d}$ fraction (lower panel)

It is interesting also to examine the profile likelihood functions for the amplitudes $N_{1i}$ characterizing the $\tilde{\chi }^0_{1}$ composition:

18 $$\begin{aligned} \tilde{\chi }^0_{1} \; = \; N_{11} {\tilde{B}} + N_{12} {\tilde{W}^3} + N_{13} {\tilde{H}_u} + N_{14} {\tilde{H}_d}, \end{aligned}$$

which are shown in Fig. 12, again for the analysis with the 13-TeV data as solid lines and without them as dashed lines, and with $(g-2)_\mu$ as blue lines and without it as green lines. The top left panel shows that, when $(g-2)_\mu$ is included, an almost pure $\tilde{B}$ composition of the $\tilde{\chi }^0_{1}$ is preferred, $N_{11} \rightarrow 1$, though the possibility that this component is almost absent is also allowed at the level $\Delta \chi ^2 \sim 4$. On the other hand, when the constraint from $(g-2)_\mu$ is removed, there is a mild ($\Delta \chi ^2 \sim 1$) preference for $N_{11} \rightarrow 0$. The reason for this is again the preference for a large $\tilde{H}_{u,d}$ components in the latter case, where the neutralino mass is allowed to be larger, due to flavor constraints slightly favoring a 1 TeV neutralino as a solution to the observed DM relic density. The upper right panel shows that a small $\tilde{W}^3$ component in the $\tilde{\chi }^0_{1}$ is preferred in all cases.^25^ Finally, the lower panel confirms that small $\tilde{H}_{u,d}$ components are preferred by $\Delta \chi ^2 \gtrsim 4$ when $(g-2)_\mu$ is included, whereas there would have been a preference for these components to dominate in the absence of the $(g-2)_\mu$ constraint.

**Fig. 13:**
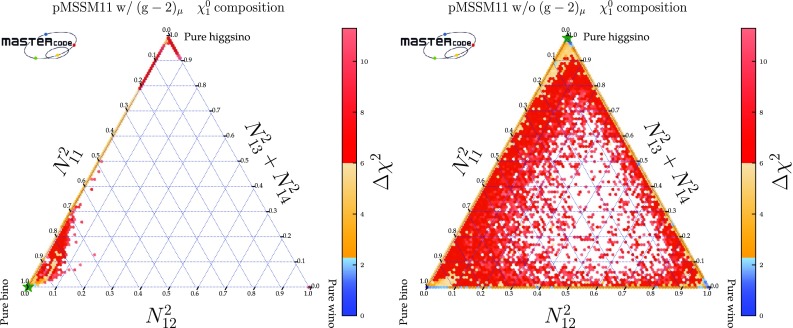
Triangular presentations of the composition of the $\tilde{\chi }^0_{1}$ in the fit with LHC 13-TeV and with (without) the $(g-2)_\mu$ constraint in the left (right) panel

Figure 13 displays information about the preferred and disfavoured $\tilde{\chi }^0_{1}$ compositions in two triangular panels. Both are for fits including LHC 13-TeV data (those dropping these data are quite similar), the left panel includes the $(g-2)_\mu$ constraint and the right panel drops it. The $\Delta \chi ^2$ for the best-fit points at each location in the triangles are colour-coded as indicated. We see in the left panel that in the case with $(g-2)_\mu$ a small Wino fraction $N_{12}^2 < 0.1$ is strongly favoured, while the relative proportions of the Bino fraction $N_{11}^2$ and the Higgsino fraction $N_{13}^2 + N_{14}^2$ are relatively unconstrained at the 95% CL. On the other hand, the right panel shows that almost all binary combinations of Bino, Wino and Higgsino (along the edges of the triangle) are allowed at the 95% CL, but three-way mixtures (in the interior of the triangle) are strongly disfavoured.

Table 6 compares the composition of the LSP $\tilde{\chi }^0_{1}$ found at the best-fit points in our present pMSSM11 analysis based on LHC 13-TeV data (with and without the $(g-2)_\mu$ constraint) with the composition at the best-fit point from our previous pMSSM10 analysis that also applied the $(g-2)_\mu$ constraint [13]. We see that both the pMSSM11 and pMSSM10 analyses with $(g-2)_\mu$ prefer an almost pure $\tilde{B}$ composition. On the other hand, when the $(g-2)_\mu$ constraint is dropped the pMSSM11 analysis prefers an almost equal mixture of $\tilde{H}_u$ and $\tilde{H}_d$ components with a small admixture of $\tilde{B}$ and again a very small admixture of $\tilde{W}_3$ because we only scan $m_{\tilde{\ell }}$ and $m_{\tilde{\tau }}$, hence $m_{\tilde{\chi }^0_{1}} < 2 \,\mathrm {TeV}$. Table 6 also displays the composition of the second-lightest neutralino, $\tilde{\chi }^0_{2}$, and we see that its content is mainly $\tilde{W}_3$ in the fit to the pMSSM11 with $(g-2)_\mu$ and in the pMSSM10 fit, but is mainly Higgsino in the fit to the pMSSM11 without $(g-2)_\mu$.

### ${\varvec{B}}$-physics observables

**Fig. 14:**
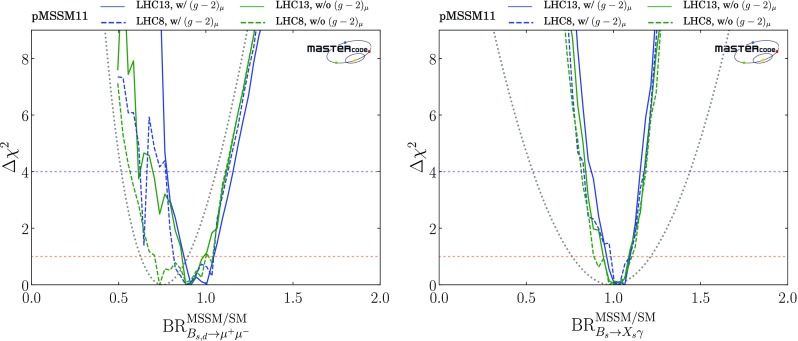
One-dimensional profile likelihood functions for $\mathrm{BR}(B_{s, d} \rightarrow \mu ^+\mu ^-)$ in the pMSSM11 (left panel) and the BR($B_s \rightarrow X_s \gamma$) branching ratio (right panel), with and without the LHC 13-TeV data and the $(g-2)_\mu$ constraint. Also shown as dotted lines are the experimental constraints, including the corresponding theoretical uncertainties within the Standard Model

Figure 14 displays the one-dimensional profile likelihood functions for $\mathrm{BR}(B_{s, d} \rightarrow \mu ^+\mu ^-)$ in the pMSSM11 (left panel) and the BR($B_s \rightarrow X_s \gamma$) branching ratio (right panel), with and without the LHC 13-TeV data and the $(g-2)_\mu$ constraint. We see in the left panel that a value of $\mathrm{BR}(B_{s, d} \rightarrow \mu ^+\mu ^-)$ close to the SM value is preferred if both these constraints are applied, though deviations at the level of $\pm \sim 10$% are allowed at the level of $\Delta \chi ^2 = 4$ (2 $\sigma$), corresponding to the 95% CL. On the other hand, if $(g-2)_\mu$ is dropped, a larger range of $\mathrm{BR}(B_{s, d} \rightarrow \mu ^+\mu ^-)$ is allowed, with a larger deviation at the level of $\pm \sim 30$% becoming allowed at the level of $\Delta \chi ^2 = 4$. In particular, when the LHC13 data are included but $(g-2)_\mu$ is dropped, the global $\chi ^2$ function is minimized at a value of $\mathrm{BR}(B_{s, d} \rightarrow \mu ^+\mu ^-)$ below the SM value, as hinted by the present experimental data, with the SM value being mildly disfavoured by $\Delta \chi ^2 \simeq 1$. It will be interesting to see how measurements of $\mathrm{BR}(B_{s, d} \rightarrow \mu ^+\mu ^-)$ evolve.

The analogous curves for BR($B_s \rightarrow X_s \gamma$) in the right panel of Fig. 14 show preferences for values close the SM predictions, with 2 $\sigma$ ranges that are $\pm 20$%. Discriminating between the SM and the pMSSM11 would require significant reductions in both the theoretical and experimental uncertainties in BR($B_s \rightarrow X_s \gamma$).

**Fig. 15:**
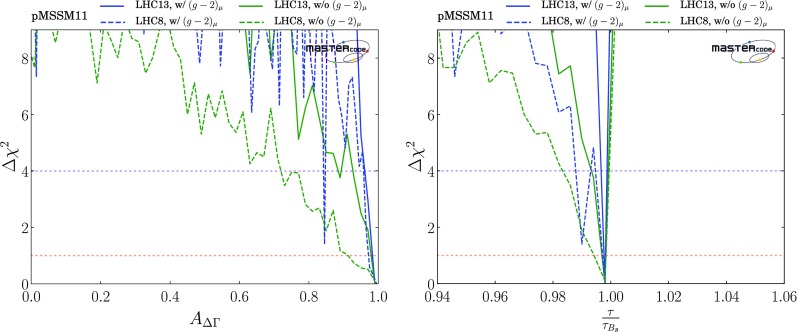
One-dimensional $\chi ^2$ profile likelihood functions for $A_{\Delta \Gamma }$ (left panel) and $\tau (B_s \rightarrow \mu ^+ \mu ^-)/\tau _{B_s}$ (right panel), in the fits with and without the LHC 13-TeV data and $(g-2)_\mu$

As already mentioned in Sect. 2.3, the LHCb Collaboration has recently announced the first experimental measurement of $\tau (B_s \rightarrow \mu ^+ \mu ^-)$, which is related to the quantity $A_{\Delta \Gamma }$ that takes the value $+ 1$ in the SM, but may be different in a SUSY model such as the pMSSM11. Figure 15 displays the profile likelihood functions for $A_{\Delta \Gamma }$ (left panel) and $\tau (B_s \rightarrow \mu ^+ \mu ^-)/\tau _{B_s}$ (right panel), in our pMSSM11 fits with and without the LHC 13-TeV data and $(g-2)_\mu$. We restrict our attention to positive values of $A_{\Delta \Gamma }$, corresponding to $\tau (B_s \rightarrow \mu ^+ \mu ^-)/\tau _{B_s} > 0.94$. We see that all the fits favour values of $A_{\Delta \Gamma }$ close to unity, with that dropping both the LHC 13-TeV data and $(g-2)_\mu$ allowing the widest range. Values of $\tau (B_s \rightarrow \mu ^+ \mu ^-)/\tau _{B_s}$ close to unity are also favoured, with $\Delta \chi ^2 \gtrsim 9$ for $\tau (B_s \rightarrow \mu ^+ \mu ^-)/\tau _{B_s} = 0.94$. The new LHCb measurement [112] does not challenge any of these model predictions.

**Table 6:** The amplitudes characterizing the decomposition of the LSP $\tilde{\chi }^0_{1}$ and of the $\tilde{\chi }^0_{2}$ into interaction eigenstates at the best-fit points in our present pMSSM11 analysis including LHC 13-TeV data, with and without the $(g-2)_\mu$constraint, compared with the composition at the best-fit point found in our previous pMSSM10 analysis that also included the $(g-2)_\mu$ constraint, but only LHC 8-TeV data [13]

Model	State	$\tilde{B}$	$\tilde{W}_3$	$\tilde{H}_u$	$\tilde{H}_d$
pMSSM11 (with $(g-2)_\mu$)	$\tilde{\chi }^0_1$	0.99	$-$0.03	0.04	$-$0.01
	$\tilde{\chi }^0_2$	0.03	0.99	$-$0.06	$-$0.01
pMSSM11 (w/o $(g-2)_\mu$)	$\tilde{\chi }^0_1$	0.01	0.04	0.71	0.70
	$\tilde{\chi }^0_2$	0.09	0.02	$-$0.70	$-$0.70
pMSSM10	$\tilde{\chi }^0_1$	0.99	$-$0.11	0.09	$-$0.04
	$\tilde{\chi }^0_2$	0.12	0.98	$-$0.13	0.05

### Higgs observables

**Fig. 16:**
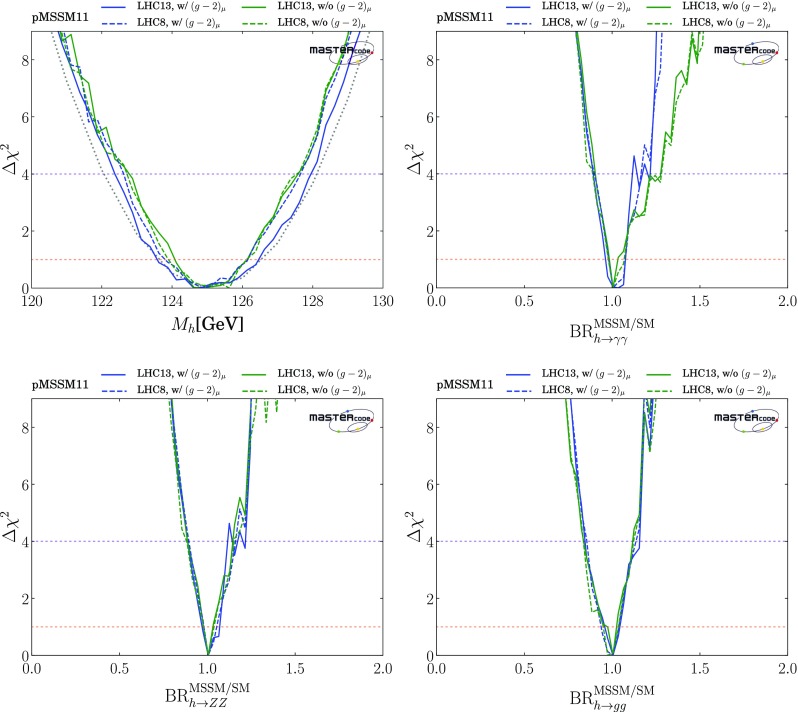
One-dimensional profile likelihood plots for $M_h$ (upper left panel), for the $h \rightarrow \gamma \gamma$ branching ratio in the pMSSM11 relative to that in the SM with (upper right panel), for the $h \rightarrow Z Z^*$ branching ratio (lower left panel) and for the $h \rightarrow gg$ branching ratio (lower right panel). In the upper left panel we also show as a dotted line the experimental constraint combined with the corresponding theoretical uncertainty within the pMSSM11

Figure 16 shows similar plots of $M_h$ (upper left panel), and of the ratios of the branching ratios for $h \rightarrow \gamma \gamma , Z Z^*$ and $h \rightarrow gg$ (treated as a proxy for $\sigma (gg \rightarrow h)$) to their values in the SM in the upper right, lower left and lower right panels, respectively. Taking into account the theoretical uncertainties in the calculation of $M_h$ in a supersymmetric model [66–71], which we take to be $\pm 3 \,\mathrm {GeV}$,^26^ there is no tension with the global fits. These also favour values of the decay branching ratios that are similar to those in the SM whether $(g-2)_\mu$ is included in the fit, or not, though with uncertainties that are typically $\pm \sim 20$%. As discussed in [34], the global combination of ATLAS and CMS measurements using LHC Run-1 data has significantly larger uncertainties.

### Dark matter measures

In Sect. 2.4 we introduced various possible mechanisms for bringing the relic $\tilde{\chi }^0_{1}$ density into the range allowed by Planck and other data, proposing measures of their prospective importance that we portrayed using different colours in the two-dimensional parameter planes shown in Sect. 3. We emphasized there and in the subsequent discussions of one-dimensional profile likelihood functions earlier in Sect. 4 the roles played by certain of these DM mechanisms. In this subsection we display profile likelihood functions for the most interesting of these DM measures, discussing the $\Delta \chi ^2$ levels at which they become relevant. As in the previous sections, we compare results for the analysis in which the $(g-2)_\mu$ constraint is applied with those when $(g-2)_\mu$ is discarded.

**Fig. 17:**
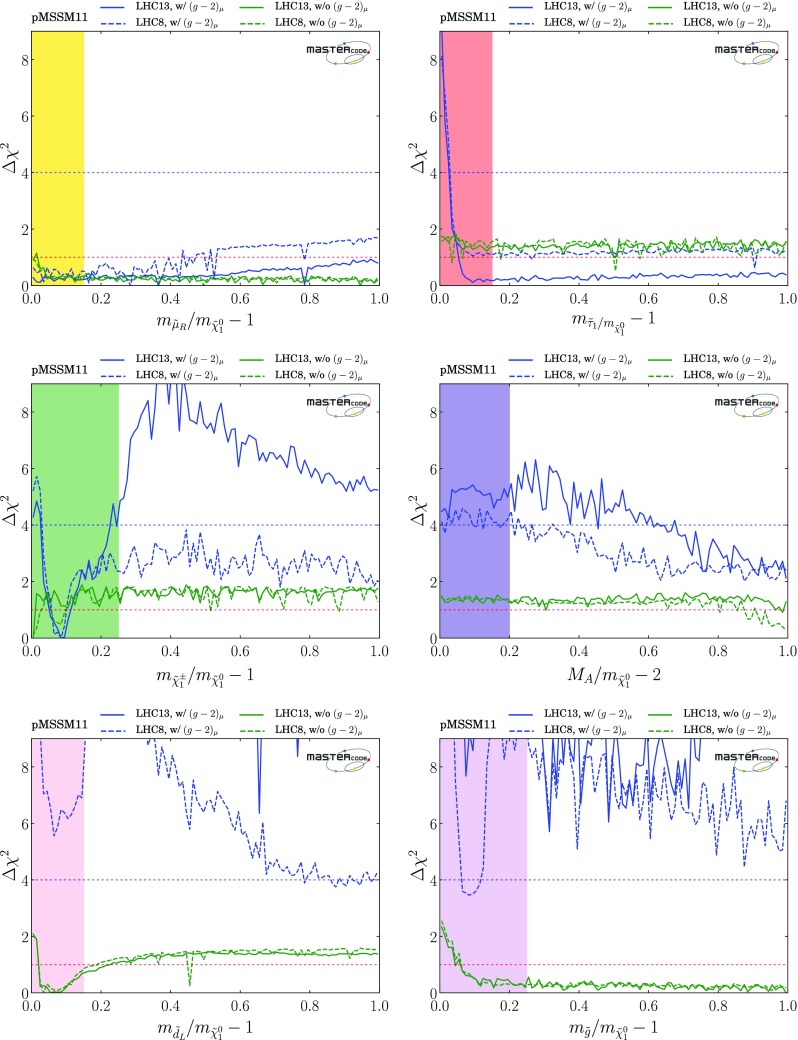
One-dimensional profile likelihood plots for the measures of the prospective importance of $\tilde{\mu }_R$ coannihilation (top left), $\tilde{\tau }_1$ coannihilation (top right), $\tilde{\chi }^\pm _{1}$ coannihilation (middle left), rapid annihilation via *A* / *H* bosons (middle right), $\tilde{d}_L$ coannihilation (bottom left) and gluino coannihilation (bottom right). The vertical coloured bands correspond to the DM mechanism criteria introduced in Sect. 2.4

Figure 17 displays the profile likelihood functions for the selected DM measures. The top left panel shows the first- and second-generation slepton measure, and we see that $\Delta \chi ^2$ is generally small throughout this region. The $\tilde{\tau }_1$ measure is shown in the top right panel, and we see that with $(g-2)_\mu$ included, whether or not the LHC 13-TeV results are included, the $\chi ^2$ function has a shallow minimum within the region where this mechanism may dominate (shown as the vertical pink band), but very small values of the $\tilde{\tau }_1$ coannihilation measure are disfavoured, and larger values of this measure also appear with a negligible likelihood price. On the other hand, when $(g-2)_\mu$ is dropped we find that $\Delta \chi ^2 \sim 2$ is almost independent of $m_{\tilde{\tau }_1}/m_{\tilde{\chi }^0_{1}}$.

The $\chi ^2$ function rises as $m_{\tilde{\tau }_1}/m_{\tilde{\chi }^0_{1}} \rightarrow 1$ when $(g-2)_\mu$ is included, because this constraint prefers small values of $m_{\tilde{\chi }^0_{1}}$, for which the relic density constraint cannot be satisfied when $m_{\tilde{\tau }_1}/m_{\tilde{\chi }^0_{1}} \rightarrow 1$. However, since the first- and second-generation slepton masses are independent of $m_{\tilde{\tau }_1}$ in the pMSSM11 there is no such obstacle disfavouring $m_{\tilde{\mu }_R}/m_{\tilde{\chi }^0_{1}} \rightarrow 1$. Therefore the profile $\chi ^2$ function for the first- and second-generation DM measure does not rise in this limit, as seen in the top left panel of Fig. 17.

In the case of the $\tilde{\chi }^\pm _{1}$ coannihilation measure shown in the middle left panel of Fig. 17, we see that the best-fit pMSSM11 points lie within this shaded band, whether the LHC 13-TeV data and/or $(g-2)_\mu$ are included or not. In the case with $(g-2)_\mu$, the best-fit point has $m_{\tilde{\chi }^\pm _{1}}/m_{\tilde{\chi }^0_{1}} \sim 1.1$ whether the LHC 13-TeV data are included or not, whereas when $(g-2)_\mu$ is dropped there is a strong preference for $m_{\tilde{\chi }^\pm _{1}}/m_{\tilde{\chi }^0_{1}}$ close to unity, which is possible in the case because the LSP is Higgsino-like. As in the case of the $\tilde{\tau }_1$ DM measure, the relic density constraint disfavours $m_{\tilde{\chi }^\pm _{1}}/m_{\tilde{\chi }^0_{1}} \rightarrow 1$ when $(g-2)_\mu$ is included. We find some parameter sets with $m_{\tilde{\chi }^\pm _{1}}-m_{\tilde{\chi }^0_{1}} \lesssim 10 \,\mathrm {MeV}$ that have $\Delta \chi ^2 \gtrsim 4$, which occur when $M_1$ is negative, near the border of a region where the LSP would be the $\tilde{\chi }^\pm _{1}$.

In the case of the *A* / *H* measure shown in the middle right panel, we see that $\Delta \chi ^2 > 3$ in this region when the $(g-2)_\mu$ and LHC 13-TeV constraints are both used. However, the $\chi ^2$ price of rapid annihilation through the *A* / *H* poles is reduced if either of these constraints is dropped. Indeed, including the $(g-2)_\mu$ constraint forces the neutralino mass to be at most $\simeq 500$ GeV, in which case the funnel condition implies an upper bound on $M_A \lesssim 1~\mathrm {TeV}$, well within the reach of LHC 13-TeV searches for $\tan \beta \gtrsim 15$.

The bottom left panel of Fig. 17 displays the profile likelihood function for the squark coannihilation measure $m_{\tilde{q}_L} /m_{\tilde{\chi }^0_{1}} - 1$. We see that before the LHC-13 data the best-fit point with $(g-2)_\mu$ included was in the squark coannihilation region with $m_{\tilde{q}_L} /m_{\tilde{\chi }^0_{1}} < 1.1$, though this feature was absent when $(g-2)_\mu$ was dropped. Including the LHC 13-TeV data, the best-fit points with and without $(g-2)_\mu$ have $m_{\tilde{q}_L} \gg m_{\tilde{\chi }^0_{1}}$, but there is still a vestige of the squark coannihilation region with $\Delta \chi ^2 < 4$ when $(g-2)_\mu$ is dropped. The reason for this is that lifting the $(g-2)_\mu$ constraint allows for a heavier neutralino, which in turn implies heavier squark masses still allowed by LHC-13 TeV data Finally, the bottom right panel of Fig. 17 shows the gluino coannihilation measure, and we see that this may also play a role when $\Delta \chi ^2 < 4$, unless both the LHC 13-TeV data and $(g-2)_\mu$ are included.

### NLSP lifetimes

**Fig. 18:**
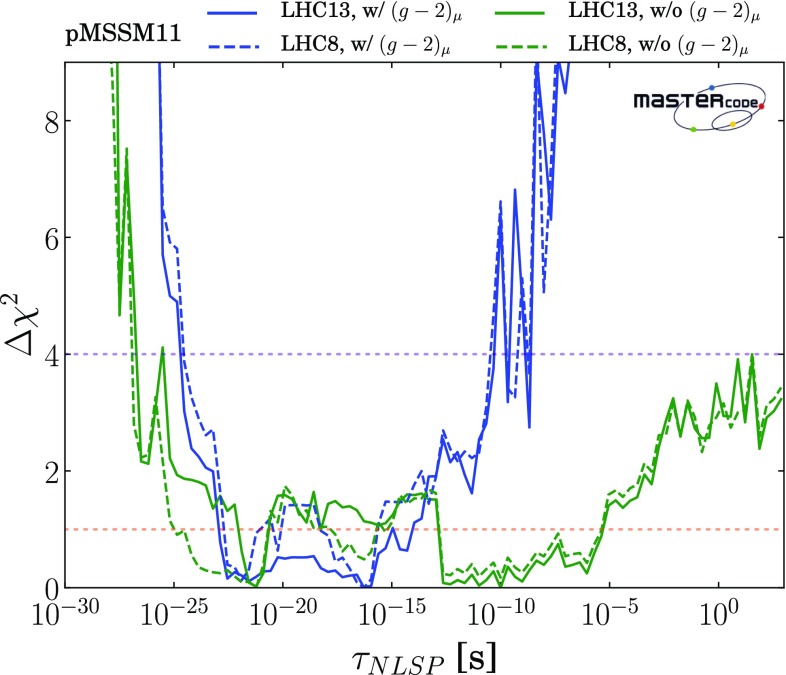
One-dimensional profile likelihood plot for the NLSP lifetime, $\tau _\mathrm{NLSP}$, including all possible NLSP species

We display in Fig. 18 the one-dimensional profile likelihood for the NLSP lifetime, $\tau _\mathrm{NLSP}$, including all possible NLSP species. There is little difference between the $\Delta \chi ^2$ functions with $(g-2)_\mu$, whether or not the LHC 13-TeV data are included (blue curves). In both cases, we find that $\Delta \chi ^2 \gtrsim 4$ for $\tau _\mathrm{NLSP} \gtrsim 10^{-10}$ s. On the other hand, when the $(g-2)_\mu$ constraint is dropped (green curves), we see that values of $\tau _\mathrm{NLSP} \lesssim 10^3$ s are allowed at the $\Delta \chi ^2 \lesssim 4$ level, again whether or not the LHC 13-TeV data are included (green curves). As already mentioned, we exclude from our scan parameter sets with NLSP lifetimes exceeding $10^3$ s, as they could alter the successful predictions of standard Big Bang nucleosynthesis [153–159].

**Fig. 19:**
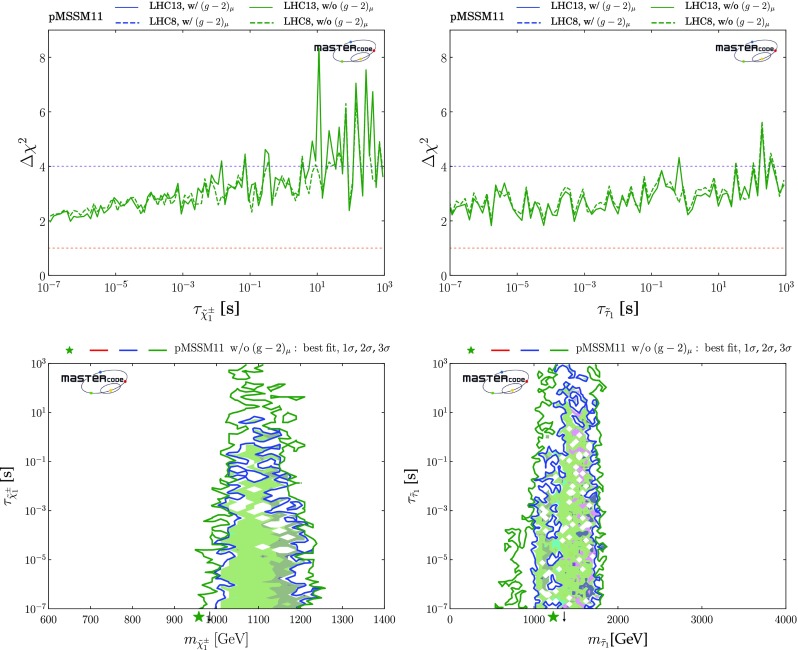
Upper panels: One-dimensional profile likelihood plots for the lifetime of the $\tilde{\chi }^\pm _{1}$ (left) and the $\tilde{\tau }_1$ (right). Lower panels: The corresponding mass-lifetime planes for the $\tilde{\chi }^\pm _{1}$ and $\tilde{\tau }_1$, with the 95% CL regions shaded according to the dominant DM mechanisms

The upper panels of Fig. 19 display the $\Delta \chi ^2$ distributions for chargino (left) and stau lifetimes (right) between $10^{-7}$ s and $10^3$ s, for the fits omitting $(g-2)_\mu$ (fits including $(g-2)_\mu$ give $\Delta \chi ^2$ outside the displayed range). We see that, whereas shorter lifetimes are favoured, lifetimes as long as $10^3$ s are allowed at the 95% CL for both sparticle species when $(g-2)_\mu$ is dropped, whether or not the LHC 13-TeV data are included. The lower panels of Fig. 19 display the corresponding mass-lifetime planes for the chargino and stau. We see that a long-lived chargino would have a mass $m_{\tilde{\chi }^\pm _{1}} \sim 1.1 \,\mathrm {TeV}$, and a long-lived stau would have a mass $m_{\tilde{\tau }_1}\sim 1.5 \,\mathrm {TeV}$, both beyond the reaches of current LHC searches for long-lived charged particles. We have also checked the possible lifetimes of other NLSP candidates, finding that squarks and gluinos generally have lifetimes $\lesssim 10^{-17} (10^{-10})$ s at the 95% CL in fits including LHC 13-TeV with (without) the $(g-2)_\mu$ constraint, with just a few points having longer lifetimes. Hence they also do not offer good prospects for LHC searches for long-lived particles.

### Spin-independent scattering cross section

**Fig. 20:**
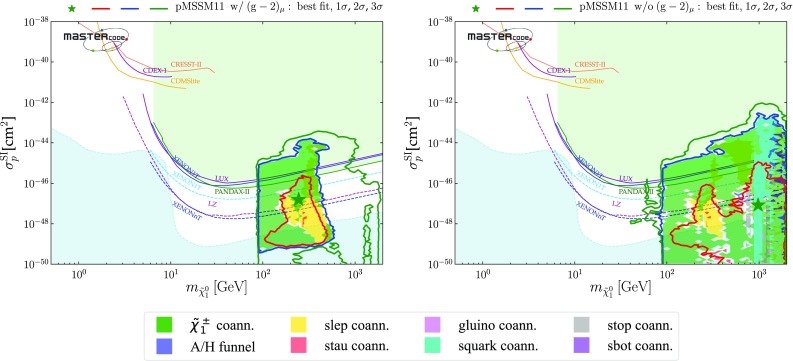
Planes of $(m_{\tilde{\chi }^0_{1}}, \sigma ^\mathrm{SI}_p)$ with (left panel) and without (right panel) the $(g-2)_\mu$ constraint applied, where the values of $\sigma ^\mathrm{SI}_p$ displayed are the nominal values calculated using the SSARD code. The upper limits established by the LUX [4], XENON1T [6] and PandaX-II [3] Collaborations are shown as green, magenta and blue contours, respectively, and the combined limit is indicated by a black line with green shading above. The projected future 90% CL exclusion sensitivities of the LUX-Zeplin (LZ) [168] and XENON1T/nT [169] experiments are shown as solid purple and dashed blue lines, respectively, and the neutrino background ‘floor’ is shown as a dashed light-blue line with a shading of the same colour below

We now discuss the prospects for direct detection of $\tilde{\chi }^0_{1}$ DM via spin-independent elastic scattering. Fig. 20 shows $(m_{\tilde{\chi }^0_{1}}, \sigma ^\mathrm{SI}_p)$ planes, including the LHC 13-TeV data, with (left panel) and without (right panel) the $(g-2)_\mu$ constraint. The values of $\sigma ^\mathrm{SI}_p$ displayed are the nominal values calculated using the central values of the matrix elements in the SSARD code. The pale green shaded region is that excluded by the combined LUX [4], XENON1T [6] and PandaX-II [3] limit, which is shown as a solid black line.^27^ The yellow shaded region lies below the neutrino ‘floor’, which is shown as an orange dashed line. We see that $m_{\tilde{\chi }^0_{1}} \gtrsim 100 \,\mathrm {GeV}$ in both the cases with and without the $(g-2)_\mu$ constraint, with upper limit $m_{\tilde{\chi }^0_{1}} \lesssim 550$ at the 95% CL when $(g-2)_\mu$ is included. When this constraint is dropped, the 95% CL range extends up to $2 \,\mathrm {TeV}$, the upper limit for which our analysis is applicable, because we have limited our scan to slepton masses $\le 2 \,\mathrm {TeV}$.

We see that the nominal prediction for $\sigma ^\mathrm{SI}_p$ at the best-fit point is at the level of the sensitivities projected for the planned LUX-Zeplin (LZ) and XENON1T/nT experiments (solid purple line) when the $(g-2)_\mu$ constraint is dropped, and somewhat higher if $(g-2)_\mu$ is included. However, we emphasize that there are considerable uncertainties in the estimate of $\sigma ^\mathrm{SI}_p$, which are reflected in the fact that the range of nominal SSARD predictions extends above the current combined limit from the LUX [4], XENON1T [6] and PandaX-II [3] experiments. There is no incompatibility when the uncertainties in the $\sigma ^\mathrm{SI}_p$ estimate are taken into account. The 68 and 95% CL ranges of the nominal values of $\sigma ^\mathrm{SI}_p$ extend slightly below the neutrino ‘floor’ in the case with $(g-2)_\mu$ included, and much lower in the case where $(g-2)_\mu$ is dropped. In both cases, large values of $\sigma ^\mathrm{SI}_p$ occur in the chargino coannihilation region (green shaded area), with other DM mechanisms including squark coannihilation yielding large values of $\sigma ^\mathrm{SI}_p$ for $m_{\tilde{\chi }^0_{1}} \gtrsim 1 \,\mathrm {TeV}$. However, this and the other DM mechanisms indicated also allow much smaller values of $\sigma ^\mathrm{SI}_p$. As in the case of the pMSSM10 studied in [13], we expect that points with very small values of $\sigma ^\mathrm{SI}_p$ would, in general, have similarly small values for the spin-independent scattering cross section on neutrons.

### Spin-dependent scattering cross section

**Fig. 21:**
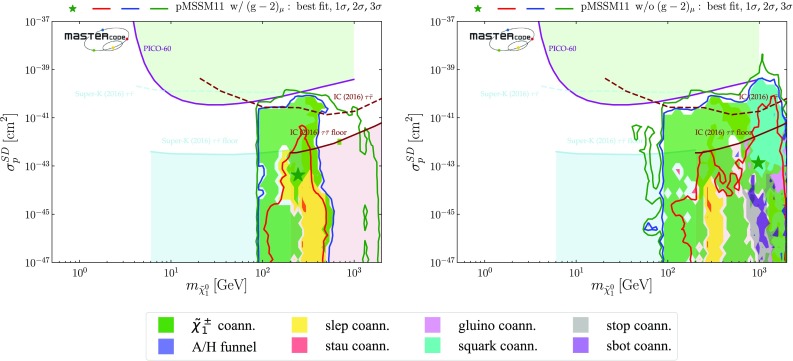
Planes of $(m_{\tilde{\chi }^0_{1}}, \sigma ^\mathrm{SD}_p)$ with (left panel) and without (right panel) the $(g-2)_\mu$ constraint applied, where the values of $\sigma ^\mathrm{SD}_p$ displayed are the nominal values calculated using the SSARD code [85]. The upper limit established by the PICO Collaboration [5] is shown as a purple contour, with green shading above. The neutrino ‘floor’ for $\sigma ^\mathrm{SD}_p$ is taken from [170]. We also show the indicative upper limits from SuperKamiokande [171] and IceCube [141] searches for energetic solar neutrinos obtained assuming that the LSPs annihilate predominantly into $\tau ^+ \tau ^-$, which are subject to the caveats discussed in the text

Figure 21 displays the corresponding planes of $(m_{\tilde{\chi }^0_{1}}, \sigma ^\mathrm{SD}_p)$ with (left panel) and without (right panel) the $(g-2)_\mu$ constraint applied. Here the neutrino ‘floor’ is taken from [170]. As in the $\sigma ^\mathrm{SI}_p$ case, we see that the allowed ranges of $m_{\tilde{\chi }^0_{1}}$ extend from $\sim 100 \,\mathrm {GeV}$ to $\sim 550 \,\mathrm {GeV}$ when $(g-2)_\mu$ is included and up to the sampling limit of $2 \,\mathrm {TeV}$ when $(g-2)_\mu$ is dropped. The uncertainties in the calculation of $\sigma ^\mathrm{SD}_p$ are significantly smaller than those for $\sigma ^\mathrm{SI}_p$, and we see that the ranges of the 68 and 95% regions in the nominal $\sigma ^\mathrm{SD}_p$ calculations lie below the upper limit from the PICO experiment [5] (solid purple line). In both the left and right panels, the nominal predictions for the best-fit points lie some $\sim 3$ orders of magnitude below the current PICO limit. For completeness, we also show the upper limits from SuperKamiokande [171] and IceCube [141] searches for energetic solar neutrinos, assuming that the LSPs annihilate predominantly into $\tau ^+ \tau ^-$ (which is not always the case in the pMSSM11) and neglecting the uncertainties in interpretation mentioned earlier: see the discussion in the following section.

**Fig. 22:**
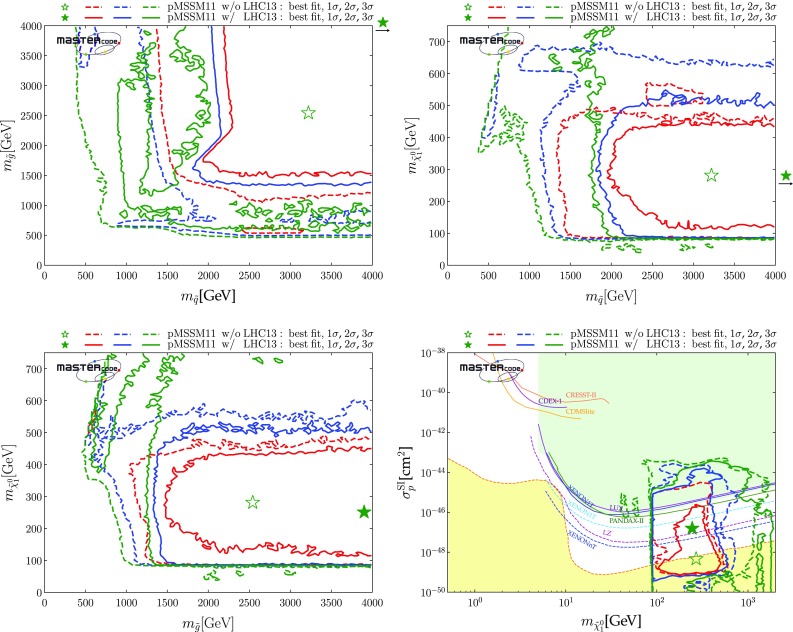
Two-dimensional projections of the global likelihood function for the pMSSM11 in the $(m_{\tilde{q}}, m_{\tilde{g}})$ and $(m_{\tilde{q}}, m_{\tilde{\chi }^0_{1}})$ planes (upper panels) and the $(m_{\tilde{g}}, m_{\tilde{\chi }^0_{1}})$ and $(m_{\tilde{\chi }^0_{1}}, \sigma ^\mathrm{SI}_p)$ planes (lower panels). The plots compare the regions of the pMSSM11 parameter space favoured at the 68% (red lines), 95% (blue lines) and 99.7% CL (green lines) in a global fit including the LHC 13-TeV data and recent results from the Xenon-based direct detection experiments LUX, XENON1T, and PandaX-II [3, 4, 6] (solid lines), and omitting them (dashed lines)

**Fig. 23:**
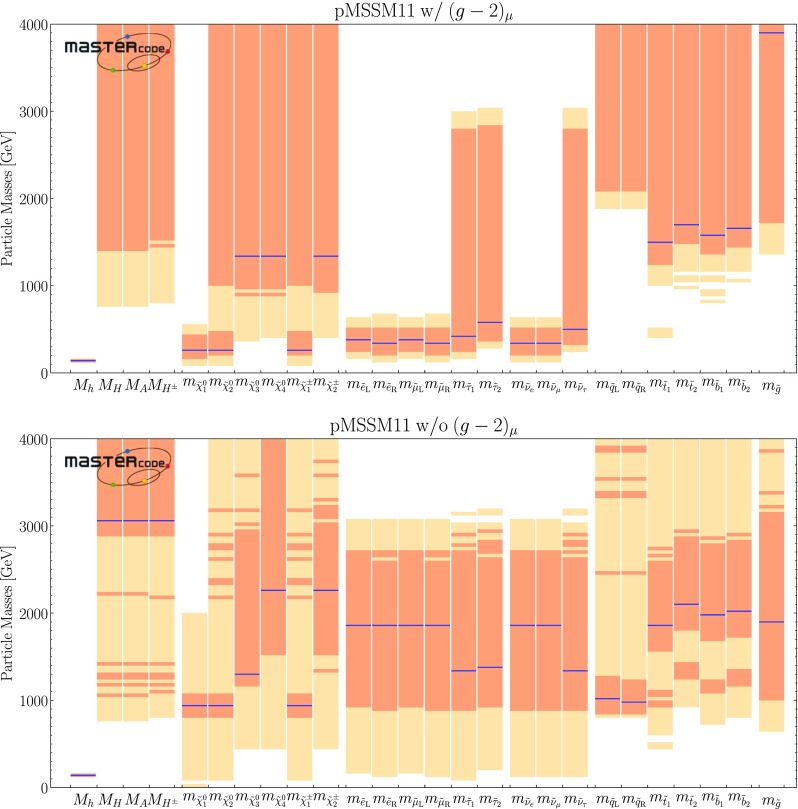
Higgs and sparticle spectrum for the pMSSM11 with and without the $(g-2)_{\mu }$ constraint applied (upper and lower panels, respectively). The values at the best-fit points are indicated by blue lines, the 68% CL ranges by orange bands, and the 95% CL ranges by yellow bands

**Fig. 24:**
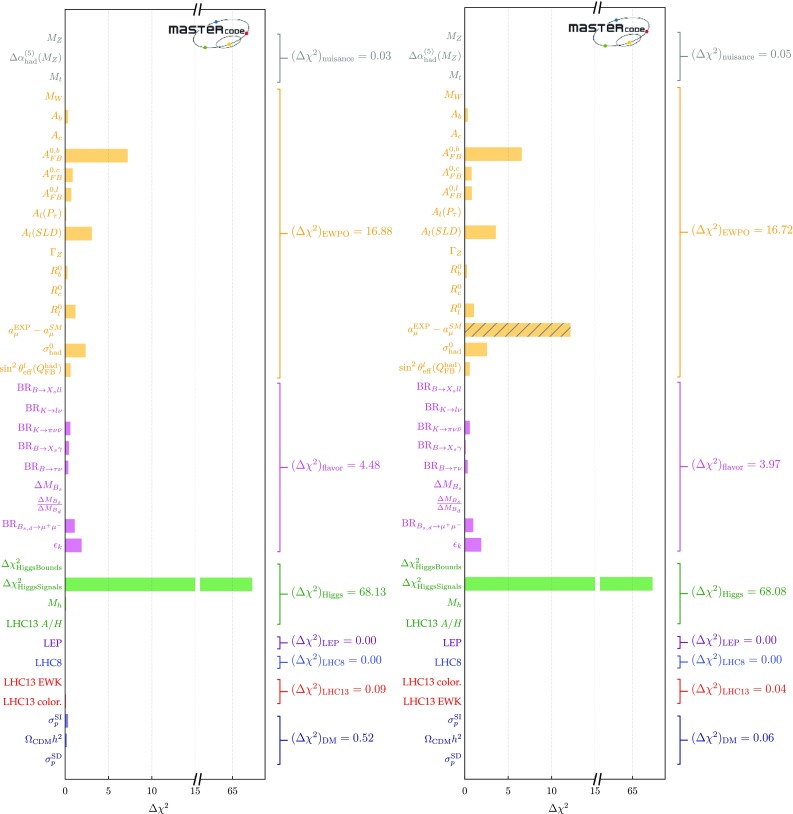
The $\chi ^2$ pulls at the best-fit points in the pMSSM11 including (left) and without the $(g-2)_\mu$ constraint (right). In the rightmost plot, the $\chi ^2$ pull from $(g-2)_\mu$ is shown (hatched orange bar), but its penalty is not included in the fit

We see in the left panel of Fig. 21 (when $(g-2)_\mu$ is included) that points with chargino coannihilation as the dominant DM mechanism yield nominal predictions for $\sigma ^\mathrm{SD}_p$ that extend over many orders of magnitude below the current PICO limit and well below the $\tau ^+\tau -$ floor. Points for which slepton coannihilation is the dominant DM mechanism do not reach so close to the PICO limit, but may also lie many orders of magnitude below it. We see in the right panel (when $(g-2)_\mu$ is dropped) similar ranges of nominal $\sigma ^\mathrm{SD}_p$ values. We also see that when $m_{\tilde{\chi }^0_{1}} \gtrsim 1 \,\mathrm {TeV}$ many competing DM mechanisms come into play, and may give small values of $\sigma ^\mathrm{SD}_p$. However, in the case of squark coannihilation $\sigma ^\mathrm{SD}_p$ may lie within $\sim 3$ orders of magnitude of the PICO upper limit.

### Indirect astrophysical searches for dark matter

We have explored the possible impact of indirect searches for DM via annihilations into neutrinos inside the Sun. If the DM inside the Sun is in equilibrium between capture and annihilation, the annihilation is quadratically sensitive to the local Galactic DM density. However, as discussed earlier, equilibrium is not always a good approximation. We note also that the capture rate is not determined solely by spin-dependent scattering on protons in the Sun, but also depends on the amount of spin-independent scattering on Helium and heavy nuclei. As we have seen, the $\sigma ^\mathrm{SI}_p$ matrix element is more uncertain than that for $\sigma ^\mathrm{SD}_p$, and this uncertainty should be propagated into the constraint on $\sigma ^\mathrm{SD}_p$. Finally, we note that the greatest sensitivity of the IceCube search for energetic neutrinos from the Sun [141] is for annihilations into $\tau ^+ \tau ^-$ and $W^+ W^-$, which are not always the dominant final states in the pMSSM11 models of interest.

Using the nominal values of the matrix elements from SSARD and neglecting the astrophysical uncertainties, we have calculated the signals in the IceCube detector for a subset of our pMSSM11 points that are consistent with the PICO constraint [5]. We find that the IceCube $W^+ W^-$ constraint [141] has negligible impact on these parameter sets, and that only a fraction are affected by the IceCube $\tau ^+ \tau ^-$ constraint. In view of this and the uncertainties in the interpretation of the IceCube searches, we have not included them in our fits.

## Impacts of the LHC 13-TeV and new direct detection constraints

In this section we illustrate the impact of the LHC 13-TeV data and the recent updates from the Xenon-based direct detection experiments LUX, XENON1T, and PandaX-II [3, 4, 6] on relevant pMSSM11 parameter planes. In the left panel of Fig. 22 we display the impact of the new results on the $(m_{\tilde{q}}, m_{\tilde{g}})$ plane: the solid red, blue and green lines are the current 68%, 95% and 99.7% CL contours, and the dashed lines are those for the corresponding 68, 95% and 99.7% CL contours in a global fit omitting the LHC 13-TeV constraints and those from the Xenon-based direct detection experiments. The right panel of Fig. 22 makes a similar comparison of the 68, 95 and 99.7% CL regions in the $(m_{\tilde{\chi }^0_{1}}, \sigma ^\mathrm{SI}_p)$ plane found in global fits including LHC 13-TeV and Xenon-based detector data (solid lines) and omitting these data (dashed lines).

We see in the upper left panel of Fig. 22 that the LHC 13-TeV constraints exclude bands of parameter space at low $m_{\tilde{q}}$ and $m_{\tilde{g}}$, disallowing in particular a squark coannihilation region at $m_{\tilde{q}}\sim 500 \,\mathrm {GeV}$ and large $m_{\tilde{g}}$ and a gluino coannihilation strip at $m_{\tilde{g}}\sim 500 \,\mathrm {GeV}$ that were allowed by the LHC 8-TeV data. The impact on the gluino and squark coannihilation strips can also be appreciated from the upper right and lower left panels, where they appear as dashed-blue islands along the diagonal where the mass is degenerate with the neutralino that disappear completely after the inclusion of the LHC 13-TeV constraints. The bottom right panel of Fig. 22 shows that low values of $\sigma ^\mathrm{SI}_p$ that would have been allowed in a fit without the LHC 13-TeV data are now disallowed. This effect is in addition to the downwards pressure on $\sigma ^\mathrm{SI}_p$ exerted by the new generation of Xenon-based direct detection experiments.

## Best-fit points, spectra and decays

Following our previous discussions of some two-dimensional projections of the pMSSM11 parameter space and various one-dimension profile likelihood functions, we now discuss in more detail the best-fit points in the pMSSM11 fits incorporating the LHC 13-TeV data, both with and without the $(g-2)_\mu$ constraint, whose input pMSSM11 parameter values were given in the first and third columns of Table 4. We note, however, that the likelihood functions are very flat for larger masses, so these best-fit points should not be taken as definite predictions.

Figure 1 displays the spectra of Higgs bosons and sparticles at the best-fit points for the pMSSM11 including (upper panel) and excluding (lower panel) the $(g-2)_\mu$ constraint.^28^ In each case we also show the decay paths with branching ratios $> 5\%$, the widths of the lines being proportional to the branching ratios. The heavier Higgs bosons $H, A, H^\pm$, are lighter in the case without $(g-2)_\mu$, whereas the sleptons and the electroweak inos are heavier. The branching ratio patterns differ in the two cases, with the Higgs bosons mainly decaying to SM particles when $(g-2)_\mu$ is not imposed. We note that the first- and second-generation sleptons are much lighter than the third-generation sleptons in the case with $(g-2)_\mu$. The third-generation squarks are also heavier when $(g-2)_\mu$ is dropped, whereas the gluino and the first- and second-generation squarks are lighter in this case. In both cases, the third-generation squarks may lie within reach of future LHC runs, whereas the first- and second generation squarks would be accessible only if $(g-2)_\mu$ is dropped. The gluino would also be accessible in this case, and possibly also if $(g-2)_\mu$ is included.

We re-emphasize that the remarks in the previous paragraph apply to the best-fit points, and that the spectra might differ significantly, as the likelihood functions are quite flat for large masses. The 68 and 95% CL ranges are displayed in Fig. 23 as orange and yellow bands, respectively, with the best-fit values indicated by blue lines. We see that for most sparticles the 95 and even 68% CL ranges extend into the ranges accessible to future LHC runs. As was to be expected, the best prospects for measuring sparticles at a linear $e^+ e^-$ collider such as ILC [172, 173] or CLIC [174] are offered by first- and second-generation sleptons and the lighter electroweak inos $\tilde{\chi }^0_{1}, \tilde{\chi }^0_{2}$ and $\tilde{\chi }^\pm _{1}$ in the case with the $(g-2)_\mu$ constraint applied.

Figure 24 displays the breakdowns of the global $\chi ^2$ functions in the cases with (left panel) and without (right panel) the $(g-2)_\mu$ constraint.^29^ The different classes of observables are grouped together and colour-coded. We see that $M_W$ makes only a small contribution, and that the total contribution to the global $\chi ^2$ function of the precision electroweak observables are quite similar in the two cases. The total contribution of the flavour sector is slightly reduced when $(g-2)_\mu$ is dropped: $\Delta \chi ^2 \sim - 1.2$, largely because of a better fit to $\mathrm{BR}(B_s \rightarrow \mu ^+\mu ^-)$, but this improvement is not very significant. The contributions of the Higgs, LEP, LHC and DM sectors are again very similar in the fits with and without $(g-2)_\mu$.

## Conclusions

In this paper we have used the MasterCode tool to analyze the constraints on the parameter space of the pMSSM11 model, in which the soft SUSY-breaking contributions to the masses of the first- and second-generation sleptons are allowed to vary independently from the third-generation slepton mass. We have taken into account the available constraints on strongly- and electroweakly-interacting sparticles from $\sim 36/\hbox {fb}$ of LHC data at 13 TeV [14–16] and the most recent limits from the LUX, PICO, XENON1T and PandaX-II experiments [3–6] searching directly for DM scattering. In addition, we have updated the constraint from the measurement of $M_W$ and some constraints from flavour observables, as described in Table 2. We have presented the results from two global fits, one including the $(g-2)_\mu$ constraint and without it. We have also made various comparisons with fits without the LHC 13-TeV data. Comparing with our earlier fit to the pMSSM10 [13], we note that the freedom for $m_{\tilde{\ell }}\ne m_{\tilde{\tau }}$ plays an important role in best fits. Furthermore, there is a big difference between $M_1$ and $M_2$ at the best-fit point without $(g-2)_\mu$.

The most visible impact of the LHC 13-TeV constraints has been on the masses of the strongly-interacting sparticles: see the left panels of Figs. 8 and 9 and compare the solid and dashed curves. On the other hand, the impact of the LHC constraints on electroweak inos has been less marked: see Fig. 11. As was to be expected, the importance of the $(g-2)_\mu$ constraint is seen in the likelihood functions for charged slepton masses and electroweak inos: compare the blue and green curves in Figs. 10 and 11. The composition of the LSP $\tilde{\chi }^0_{1}$ is also different in the cases with and without $(g-2)_\mu$: as seen in Fig. 12 and Table 6, a $\tilde{B}$ LSP is preferred when $(g-2)_\mu$ is included, whereas a $\tilde{H}$ LSP is preferred when $(g-2)_\mu$ is dropped. Moreover, the inclusion of the $(g-2)_\mu$ constraint also has significant indirect implications for the squark masses, as also seen in Figs. 8 and 9. This analysis reinforces the importance of clarifying the interpretation of the difference between the experimental measurement and the SM calculation of $(g-2)_\mu$. We therefore welcome the advent of the Fermilab $(g-2)_\mu$ experiment [175] and continued efforts to refine the SM calculation.

We have also analyzed in this paper the importances of different mechanisms for bringing the relic LSP density into the range favoured by Planck 2015 and other data: see the shadings in Figs. 2, 4, 5, 6, 19, 20 and 21, and the profile $\chi ^2$ functions for the DM measures in Fig. 17. As we see there, important roles are played by chargino coannihilation, slepton coannihilation and rapid annihilation via direct-channel *H* / *A* boson exchange, though other mechanisms such as stau and squark coannihilation may be important in limited regions of parameter space.^30^ In the case where the $(g-2)_\mu$ constraint is dropped, there is a preference for a region where $m_{\tilde{\chi }^0_{1}} \sim m_{\tilde{\chi }^\pm _{1}} \sim m_{\tilde{q}}\sim m_{\tilde{g}}$ where multiple coannihilation processes play a role, and the compressed spectrum reduces the sensitivity of the LHC sparticle searches.

In general, our analysis favours quite small deviations from the SM predictions for electroweak, flavour and Higgs observables: see Figs. 12 and 14, in particular. We have also analyzed the pMSSM11 predictions for the $A_{\Delta \Gamma }$ and $\tau (B_s \rightarrow \mu ^+ \mu ^-)$ observables recently measured for the first time by the LHCb Collaboration [112]. As seen in Fig. 13, the pMSSM11 predictions for these observables are very similar to those in the SM, deviating by much less than the current experimental uncertainties. Accordingly, we do not include $A_{\Delta \Gamma }$ and $\tau (B_s \rightarrow \mu ^+ \mu ^-)$ in our global fits.

We find that current LHC searches for long-lived particles do not impact our scan of the pMSSM11 parameter space. However, the pMSSM11 still offers significant prospects for the discovery of long-lived particles. When the $(g-2)_\mu$ constraint is imposed, we find that $\Delta \chi ^2 \gtrsim 4$ for $\tau _\mathrm{NLSP} \gtrsim 10^{-10}$ s. However, when the $(g-2)_\mu$ constraint is dropped, values of $\tau _\mathrm{NLSP}$ as long as $10^3$ s (the limit we impose in order to maintain successful Big Bang nucleosynthesis) are allowed at the $\Delta \chi ^2 \lesssim 4$ level,

As seen in Figs. 20 and 21, the pMSSM11 offers interesting prospects for the detection of supersymmetric DM. In both the spin-independent and -dependent cases, cross sections close to the present experimental upper limits are favoured at the 68% CL, whether or not $(g-2)_\mu$ is included in the set of constraints. Interestingly, in the case of $\sigma ^\mathrm{SI}_p$ with $(g-2)_\mu$ included, there is a lower limit that is not far below the neutrino ‘floor’,^31^ whereas $\sigma ^\mathrm{SI}_p$ may be much lower when $(g-2)_\mu$ is dropped, and low values of $\sigma ^\mathrm{SD}_p$ are allowed in both cases.

We turn finally to the prospects for discovering sparticles in future runs of the LHC, or with a future linear $e^+ e^-$ collider. As seen in Fig. 21, whether or not $(g-2)_\mu$ is included in the global fit, the third-generation squarks may well be within reach of future LHC runs, and the first- and second-generation squarks and the gluino may also be accessible if the $(g-2)_\mu$ constraint is dropped. If it is included, on the other hand, there are also good prospects for discovering electroweakly-interacting sparticles at an $e^+ e^-$ collider, in particular the ${\tilde{e}}, {\tilde{\mu }}, \tilde{\chi }^0_{1}, \tilde{\chi }^0_{2}$ and $\tilde{\chi }^\pm _{1}$.

It is often said that the night is darkest just before dawn, and the same may be true for supersymmetry.

## Footnotes

1 We use here results from SUSY searches by the CMS Collaboration: the results from ATLAS [2] yield similar constraints.

2 For a recent analysis of these models in light of $\sim 13$/fb of LHC data at 13 TeV, see [32]. This analysis does not include the PICO, XENON1T and most recent PandaX-II results, and has other differences that are noted later in this paper.

3 For a recent analysis of a 7-dimensional version of the MSSM in light of $\sim 13$/fb of LHC data at 13 TeV, see [52].

4 In comparison, the pMSSM7 scenario studied in [52] assumes gaugino and squark/slepton mass universality at some input scale *Q*, and has two trilinear couplings $A_{t,b}$, independent Higgs masses $H_{u,d}$ and $\tan \beta$ as free parameters.

5 Since $m_{\tilde{\ell }}> m_{\tilde{\chi }^0_{1}}$, this entails also the restriction to $m_{\tilde{\chi }^0_{1}} < 2 \,\mathrm {TeV}$ visible in subsequent figures.

6 We use here an updated version of FeynWZ (not yet publicly available) in which the $M_W$ evaluation is based on [65] and is identical to that implemented in FeynHiggs, which gives more reliable results in parameter regions with larger SUSY masses or small SUSY mass splittings. The other EWPO are treated in the same way as in [63, 64].

7 We note that FeynHiggs incorporates resummation effects in Higgs mass calculations that are not included in the MSSM FlexibleSUSY generator [72] used in [32, 52], although these are available through other FlexibleSUSY generators, HSSUSY/SplitSUSY[73–75] and FlexibleEFT[76]. It should also be noted that FeynHiggs has recently been improved for higher SUSY mass scales [77, 78].

8 The SSARD computation of the scattering cross-section follows the computations detailed in [86–88]. The uncertainties in the cross-sections are derived from a straightforward propagation of errors in the input quantities which determine the cross-section. The dominant uncertainties are discussed below in more detail.

9 We emphasize that, although they are not displayed in Table 2 because they have not changed since[34], we use a complete set of electroweak constraints, not restricted to $M_W$ as used in [32, 52]. We also note that the FeynWZ code we use to calculate $M_W$ incorporates 2-loop corrections that are not included in the FlexibleSUSY code [72] used in [32, 52].

10 In so doing, we neglect correlations in the uncertainties due to PDFs, QED and boson $p_T$ modelling, but our results are relatively insensitive to the details of this combination.

11 The $(g-2)_\mu$ evaluation in FeynHiggs contains less sophisticated two-loop corrections than GM2CALC [97]. However, the difference is small compared with other uncertainties in our analysis.

12 We refer to Table 1 of [34] for a complete set of the *K*-decay constraints we implement.

13 We have checked specifically the validity of these measures using Micromegas, finding good consistency in most cases. However, in certain hybrid regions where more than one mechanism satisfied the criteria we found that just one mechanism dominates. Moreover, it might also happen that some regions of the parameter space are not classified by a given measure even if the corresponding mechanism is active.

14 However, we note that this estimate has been challenged [130], and flag this as an issue requiring resolution.

15 For a recent estimate with a very similar central value of $\Sigma _{\pi N}$ made using covariant baryon chiral perturbation theory, see [131].

16 For comparison, a similar value of $\Sigma _{\pi N} = 59 \pm 9 \,\mathrm {MeV}$ is assumed in [32], but with $\sigma _s \equiv m_s \langle N | {\bar{s}} s | N \rangle = 43 \pm 8 \,\mathrm {MeV}$ inferred from lattice calculations. This corresponds to $\Sigma _{\pi N} - \sigma _0 = (m_u + m_d) \sigma _s/m_s \sim 3.5 \,\mathrm {MeV}$, implying a value of $\sigma _0$ different from the value we use, which is based on octet baryon masses.

17 The values (11) of the $\Delta q$ that we use are similar to those used in [32].

18 In contrast, [32] uses the IceCube result, but not the PICO result.

19 The SmodelS code [147, 148] takes a similar approach, as described in [146].

20 We note here for completeness that the LHC searches for sleptons [1, 2] do not constrain the pMSSM11 parameter space significantly.

21 In previous studies (see, e.g., the first paper in [7–10]) we have validated our naive p-value approximation with toy experiments, and found that it provides a reasonably accurate and conservative estimate of the underlying p-value of the likelihood distribution. This was confirmed by a study in the last paper in [21–31], which compared for different scenarios the naive p-value calculation with that obtained from toys.

22 This and later figures were prepared using Matplotlib [161], except where otherwise noted.

23 For relatively low stop masses, large values of $X_t/M_S \simeq A_t/M_S \simeq \sqrt{6}$ are required to avoid tension with the Higgs mass measured at the LHC. Constraints from Charged and Color Breaking (CCB) minima can be relevant [162] in such a case, but we have not taken these in account [163] in our analysis. This is because our best-fit point region is characterized by relatively small values of $A_t/M_S$, as it can be seen from Table 4, for which this issue is not relevant.

24 This effect was noted previously in a different context in [164].

25 This is because we only scan $m_{\tilde{\ell }}$ and $m_{\tilde{\tau }} < 2 \,\mathrm {TeV}$, hence $m_{\tilde{\chi }^0_{1}} < 2 \,\mathrm {TeV}$, so do not probe the expected Wino-like LSP region where $m_{\tilde{\chi }^0_{1}} \sim 3 \,\mathrm {TeV}$

26 We implement the constraint in the fit as a Gaussian likelihood-penalty with $\sigma = 1.5$ GeV, to avoid issues which would result from using a flat interval (due to the discontinuity in the p.d.f. at the interval extrema).

27 For completeness, we also show the constraints on $\sigma ^\mathrm{SI}_p$ from the CRESST-II [165], CDMSlite [166] and CDEX [167] experiments, which are most important at low values of $m_{\tilde{\chi }^0_{1}}$ that are excluded by our analysis.

28 This figure was prepared using PySLHA [160].

29 The corresponding horizontal bar has diagonal hatching, to recall that it is not included in the fit.

30 Compared to the pMSSM7 analysis in [52], we find that stop coannihilation is less prominent, and that rapid annihilation through the *Z* and the light Higgs boson is of very limited importance. In these respects the more general realization of the MSSM with four additional free parameters yields substantially different results.

31 However, we repeat that the uncertainties in the calculation of $\sigma ^\mathrm{SI}_p$ are large, and these remarks apply within the framework of a calculation of $\sigma ^\mathrm{SI}_p$ using SSARD.
